# Improved binding site assignment by high-resolution mapping of RNA–protein interactions using iCLIP

**DOI:** 10.1038/ncomms8921

**Published:** 2015-08-11

**Authors:** Christian Hauer, Tomaz Curk, Simon Anders, Thomas Schwarzl, Anne-Marie Alleaume, Jana Sieber, Ina Hollerer, Madhuri Bhuvanagiri, Wolfgang Huber, Matthias W. Hentze, Andreas E. Kulozik

**Affiliations:** 1 Department of Pediatric Oncology, Hematology and Immunology, University of Heidelberg, Im Neuenheimer Feld 430, 69120 Heidelberg, Germany; 2 Molecular Medicine Partnership Unit (MMPU), Im Neuenheimer Feld 350, 69120 Heidelberg, Germany; 3 European Molecular Biology Laboratory (EMBL) Heidelberg, Meyerhofstrasse 1, 69117 Heidelberg, Germany; 4 University of Ljubljana, Faculty of Computer and Information Science, Vecna pot 113, SI-1000 Ljubljana, Slovenia

## Abstract

Individual-nucleotide resolution crosslinking and immunoprecipitation (iCLIP) allows the determination of crosslinking sites of RNA-binding proteins (RBPs) on RNAs. iCLIP is based on ultraviolet light crosslinking of RBPs to RNA, reverse transcription and high-throughput sequencing of fragments terminating at the site of crosslinking. As a result, start sites of iCLIP fragments are expected to cluster with a narrow distribution, typically representing the site of direct interaction between the RBP and the RNA. Here we show that for several RBPs (eIF4A3, PTB, SRSF3, SRSF4 and hnRNP L), the start sites of iCLIP fragments show a fragment length-dependent broader distribution that can be shifted to positions upstream of the known RNA-binding site. We developed an analysis tool that identifies these shifts and can improve the positioning of RBP binding sites.

## Abstract (web summary)

Individual-nucleotide resolution crosslinking and immunoprecipitation (iCLIP) can map RNA binding sites of RNA-binding proteins (RBPs). Here, the authors report an analysis tool that improves the binding site assignment for some RBPs that have length-dependent broader distribution for their iCLIP fragments.

## Graphical abstract

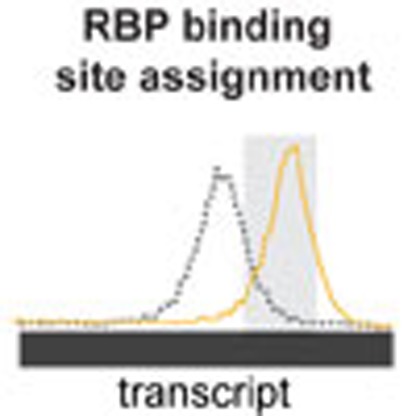

The development of crosslinking and immunoprecipitation (CLIP) combined with high-throughput sequencing (HITS) has been a milestone for the understanding of the function of ribonucleoprotein complexes in controlling gene expression^1^,^2^. CLIP relies on the ultraviolet light-induced formation of covalent bonds between RNA-binding proteins (RBPs) and the RNA, thus enabling the genome-wide and precise analysis of interactions between RNA and RBPs *in vivo*. This general principle underlies different protocols of CLIP analyses including HITS-CLIP^2^,^3^, photoactivatable-ribonucleoside-enhanced crosslinking and immunoprecipitation (PAR-CLIP^4^,^5^) and individual-nucleotide resolution crosslinking and immunoprecipitation (iCLIP^6^). In HITS-CLIP and PAR-CLIP, adapters are ligated to the fragmented RNAs both at the 5′ and at the 3′ ends. These methods therefore require read-through of the reverse transcriptase (RT) beyond the crosslinking site to reach the 5′ adaptor to enable amplification and deep-sequencing of the CLIP fragments. iCLIP is based on the frequent termination of the RT at the crosslinking site, which corresponds to the nucleotide preceding the start of the sequenced iCLIP fragment^6^ (Fig. 1). Thus, the current analyses of iCLIP data sets are based on the propensity of the RT to terminate at sites of crosslinking, generating iCLIP fragments whose sequence start sites cluster in a length-independent manner and enable mapping of the RNA crosslinking sites of the RBP with high precision.

Here we show that the iCLIP libraries of a number of RBPs contain a high proportion of fragments with non-coinciding start sites, which can impact the definition of binding sites. We describe an analysis approach that improves the binding site assignment from such iCLIP libraries.

## Results

### eIF4A3 iCLIP fragment start sites are broadly distributed

For RBPs that bind to specific sites on their target transcripts, truncation of iCLIP complementary DNAs at the crosslinking site is expected to result in clustering of the start sites of the corresponding sequenced iCLIP fragments, so that they map to a narrow region of the reference genome. To examine the truncation of iCLIP complementary DNAs in more detail, we performed iCLIP in HeLa cells with the exon junction complex (EJC) protein eIF4A3, a well-studied subunit of the EJC that directly binds to RNA^7^,^8^,^9^. We grouped the iCLIP fragments according to their lengths and distinguished the shorter fragments, whose sequences extended into the 3′ Solexa primer (group A) from the longer fragments that were not sequenced all the way through to the 3′ Solexa primer (group B; Fig. 2a). The first group thus contained fragments with a known, short length. The second group contained fragments with a longer and undefined length. It is important to note that long and short fragments do not have a different quality in mapping the correct RNA–protein interaction site *per se*. Rather, the analysis of start sites of fragments of different lengths and thus the classification into group A and B has been used as a tool which reveals that the start sites of fragments of different length do not overlap universally in all RBPs analysed. The number of fragments in group A and B, respectively, depends on the sequencing length and the conditions of the iCLIP experiment (Supplementary Fig. 1). All fragments were mapped to the reference genome using the STAR software, which allows mapping of fragments across exon–exon boundaries^10^ (Supplementary Table 1). We next plotted the distribution of fragment start and centre positions relative to specific reference points in the transcriptome (here: exon–exon junctions). These graphs are referred to as read distribution maps (Fig. 2b). Because the nucleotide preceding the start of sequenced iCLIP fragments should mark the crosslinking site of the RBP to the RNA, we expected the distribution of these start sites to be narrow, to be independent of fragment length and to centre around the known binding site of the EJC at ∼24 nucleotides (nts) 5′ of the exon–exon junction^7^,^9^,^11^,^12^. However, in contrast to these expectations, we found (1) the distribution of both, the group A and group B fragments not to peak at the known binding site of eIF4A3 but at positions that were shifted upstream, and (2) the shift of fragment start sites to be larger for the longer group B fragments (green curve) than for the shorter group A fragments (orange curve, Fig. 2b). Notably, the centre positions of the iCLIP fragments reflected the known eIF4A3 binding site at a position ∼24 nts upstream of the exon–exon junction^7^,^9^,^11^,^12^. Moreover, the width of the distribution of start sites (measured as full width at half maximum (FWHM)) across the entire population of fragments was 29 nts compared with 23 nts for the respective distribution of the centre of the fragments (Fig. 2b left and right panel, blue curves).

Because eIF4A3 is known to bind to RNA in a mostly sequence-independent manner^7^ or possibly to a low stringency conserved sequence^11^,^12^, we extended our analyses to publicly available iCLIP data sets of other RBPs with defined consensus RNA-binding sequences (Table 1). These analyses enabled us to study whether non-coinciding start sites of iCLIP fragments are a phenomenon limited to our eIF4A3 data, and if not to what extent they potentially represent a more common challenge for iCLIP analyses. Since the deposition of eIF4A3 upstream of exon–exon junctions appears to be guided by serine/arginine-rich (SR) proteins^11^, we re-analysed a previously published iCLIP data set for the splicing factor SRSF3 (ref. 13). Our re-analysis of the SRSF3 iCLIP data confirmed the reported enrichment of SRSF3 fragments upstream of exon–exon junctions, although the binding site of this protein could not be defined at high-resolution using the fragment start sites^13^ (Fig. 2c left panel). Our analysis also revealed a potential reason for this limited resolution: the start sites of the sequenced iCLIP fragments were distributed broadly (FWHM: 15 nts; Fig. 2c left panel, blue curve), reminiscent of what we first observed for eIF4A3. By contrast, the use of the fragment centres as reference points yielded a narrower distribution (FWHM: 7 nts; Fig. 2c right panel, blue curve). Interestingly, our re-analysis mapped the SRSF3 binding sites to the RNA downstream of the EJC, and not upstream of the complex, as the analysis using the start sites would have indicated. Similar results as for SRSF3, *albeit* with lower read coverage, were also obtained by re-analysing a previously published iCLIP data set for SRSF4 (Supplementary Fig. 2a)^13^.

### Start sites are broadly distributed in exons and introns

We next investigated whether the proportion of fragments with non-coinciding start sites depends on whether the RBPs bind to either exonic or intronic regions. We thus complemented the analyses of eIF4A3, SRSF3 and SRSF4, which predominantly bind to exons, with the analysis of iCLIP libraries for predominantly intron-binding proteins. To this end, we prepared iCLIP libraries for PTB and we re-analysed previously published data sets for TIAL1 (ref. 14), U2AF65 (ref. 15), hnRNP L^16^ and PTB^17^. TIAL1 binds predominantly to 5′ splice sites, whereas U2AF65, hnRNP L and PTB mostly assemble at 3′ splice sites.

The re-analyses for U2AF65 and TIAL1 showed that the start sites of sequenced fragments (1) clustered within a narrow sequence range, (2) overlapped in a manner independent of fragment length and (3) mapped the crosslinking sites to the known consensus binding sites of these RBPs. By contrast to the start sites, the centre positions of the iCLIP fragments were more unevenly distributed. This re-analysis thus confirmed the previously reported high precision iCLIP mapping of the crosslinking sites of these RBPs (Fig. 2d and Supplementary Fig. 2c). By contrast, analysis of our PTB iCLIP libraries and re-analysis of the published hnRNP L library revealed that the start sites of iCLIP fragments of different lengths did not cluster equally well. For hnRNP L, the distribution of the short fragments was narrower for the centre positions (FWHM: 7 nts; Fig. 2e right panel, orange curve) than for the start positions (FWHM: 13 nts; Fig. 2e left panel, orange curve). When using the centres of the fragments for the analysis of PTB binding data, the distribution showed that the expected enrichment was close to the 3′splice site^18^,^19^, which was not the case if the start sites were used as points of reference (Supplementary Fig. 2b).

### iCLIP fragment centres can improve binding site mapping

We next focused our analysis of eIF4A3 on the shorter completely sequenced group A fragments and generated high-resolution read distribution heatmaps, which display the start positions relative to the exon–exon junction, stratified by fragment length (Fig. 3a top panel). In these plots each row represents the start site distribution of the fragments with a defined length normalized by the total number of fragments of the respective length (the number of fragments per fragment length is shown for all RBPs analysed in Supplementary Fig. 1). According to the conventional assumptions of iCLIP, the start sites of these fragments should coincide at the crosslinking position in a length-independent manner. In contrast to this expectation, the start sites are shifted to positions upstream of the known binding site in a length-dependent manner.

We then analysed the distribution of the centre positions and observed that these clustered around the expected position of binding 24 nts upstream of the exon–exon junction^7^,^9^,^11^,^12^ (Fig. 3a middle panel). This indicates that, in situations where the start sites do not coincide in fragments of different length, the centre position is a better estimate for binding site assignment. We further complemented this high-resolution analysis by mapping the end positions of these sequenced fragments (Fig. 3a bottom panel). Considering the distribution of the fragment start sites (top panel), the distribution of the fragment ends showed the expected triangular shape. Notably, however, there was an area between 30 and 18 nts upstream of the exon–exon junctions, where neither fragment start sites nor fragment ends were localized. The paucity of fragment start sites and fragment ends in this region suggests that the RNA is protected from RNase digestion, likely representing a footprint of eIF4A3 around the expected binding position 24 nts upstream of the exon–exon junctions.

When generating a high-resolution read distribution heatmap for U2AF65, whose binding we have displayed in Fig. 2 could be mapped with high precision by standard iCLIP analysis, we found that the start sites narrowly clustered, as expected, at the known binding site 11 to 19 nts upstream of the 3′ splice site (Fig. 3b top panel). In this case, the centres and ends showed the expected broader distribution with a slope that mirrors the fragment lengths (Fig. 3b middle and bottom panel).

These data indicate that iCLIP libraries can contain different proportions of overlapping read start sites. For iCLIP libraries with fragment length-independent clustering of start sites (exemplified by U2AF65), the RBP binding site can be mapped with high-resolution by standard iCLIP analysis. For iCLIP libraries with a high proportion of non-coinciding start sites (exemplified by eIF4A3), the resolution of binding site assignment is decreased and the use of standard iCLIP analysis tools can actually result in misassignment of the binding site (see Supplementary Fig. 7 for eIF4A3 binding site assignment using different iCLIP analysis tools^6^,^20^,^21^). Thus, there is a need for analytical adjustment in iCLIP data sets of the ‘eIF4A3-type’. In these cases, the use of the fragment centre narrows down the estimated position of binding sites and improves the accuracy of the mapping of the binding sites.

### RBPs show different proportions of coinciding start sites

So far, we have considered the position of fragment start sites and centres relative to specific positions on the transcripts, which were given by their fixed position relative to splice junctions. This approach is not feasible for RBPs that are not known or expected to bind in such a fixed arrangement relative to annotated features. Therefore, we developed a tool that enables analysis of the binding profiles of RBPs independent of any annotation. We termed the graphical output of this algorithm ‘high-resolution read overlap heatmaps’. A schematic representation of the method is shown in Fig. 4a. We first applied this analysis to eIF4A3 and U2AF65. We subdivided the genome into segments of 300 bp and focused on those segments that were covered by at least 50 iCLIP reads, although these numbers can be adjusted according to the coverage of iCLIP fragments (Supplementary Fig. 1 and Supplementary Table 2). Within each segment, we defined the start, centre or end positions of the longer group B fragments, respectively, as 0-positions, which served as reference points for the heatmaps. For the analysis shown in the upper panel of Fig. 4, we calculated the offset between the start sites of each fragment of group A and the reference positions, that is, the start sites of the group B fragments in the segment. We first focused on the long fragments (group B) as a reference for the start position, because this enabled us to use the maximum number of fragments to generate high-resolution read overlap heatmaps, that is, plotting the maximum number of rows in each heatmap. Each row in the heatmap shows the histogram of the offsets (*x*-axis), stratified by fragment length as indicated on the *y*-axis. The histogram bin heights are represented by colours. For iCLIP libraries with a high proportion of coinciding start sites, the start sites of the group A fragments are expected to map to the reference positions. For iCLIP libraries with a high proportion of non-coinciding start sites, the group A fragment start sites are expected to map downstream of the reference positions, resulting in a broadening of the distribution. Indeed, the high-resolution overlap analysis showed a population of fragments with overlapping start sites for eIF4A3, but also a large proportion of fragment start sites downstream of the 0-position (Fig. 4b top panel).

By contrast, analysis of the centre positions revealed that these were more consistently distributed around the centre reference position (Fig. 4b middle panel). As expected, the overlapping start sites mapped upstream of the reference position on a diagonal with a slope that corresponded to the fragment length. However, in addition to this population of fragments with closely clustering start sites there was a large fraction of fragments whose ends mapped more closely together. In the graphical output, these fragments appear as a cloud of data points downstream of the centre reference position. Such a clustering of fragment ends was even more apparent when the ends were directly used as a reference position (Fig. 4b bottom panel).

In contrast, the start sites of U2AF65 iCLIP fragments formed a narrow peak at the 0-position with only few start sites mapping downstream (Fig. 4c top panel). Accordingly, when analysing the centre position we found that only a small number of iCLIP fragments mapped downstream of the reference position (Fig. 4c middle panel). Likewise, the end positions mostly mapped to the expected diagonal upstream of the reference, whereas a small population of fragments mapped close to the end 0-position (Fig. 4c bottom panel). Furthermore, we used different parameters that yielded different numbers of reads as an input for the read overlap analyses of eIF4A3 and U2AF65 to test the robustness of the tool (Supplementary Fig. 3 and Supplementary Tables 3 and 4). These analyses showed that the results are not confounded by the number of input reads. In addition, to these analyses we used another biological iCLIP replicate of U2AF65 that has less reads and a lower overall quality to generate a high-resolution heatmap (Supplementary Fig. 3e). The distributions are highly similar between the two biological replicates of U2AF65 and had similar proportions of overlapping start sites. Thus, even in libraries of different qualities and read numbers the proportion of overlapping start sites remains a stable property of the protein analysed and, in the example of U2AF65, favours the use of standard iCLIP analysis tools that use read start sites as a reference point for binding site assignment.

We then extended the high-resolution read overlap analysis to those proteins for which we had already generated read distribution maps (see Fig. 2 and Supplementary Fig. 2). The graphical output of the high-resolution read overlap heatmaps shows large numbers of iCLIP fragments that map downstream of the start reference position (Supplementary Fig. 4 top panels). Therefore, the use of fragment start sites assigns very wide binding sites with a bias towards a position upstream of the known binding site. The width and the offset of the assigned binding sites increased with the length of the fragments contained in the iCLIP library (see Supplementary Fig. 1). By contrast, when using the fragment centre for binding site assignment the width of the assigned binding site was reduced and the misassignment corrected, because the maximal offset from the reference to the exact binding site corresponds to half of the fragment length.

Therefore whether to use the start or centre position of the iCLIP fragments for binding site assignment is an important question. The graphical outputs of the high-resolution overlap analyses of different iCLIP libraries show a significant degree of variability of non-overlapping start sites (Fig. 4 and Supplementary Fig. 4). In principle, in iCLIP libraries with predominantly overlapping start sites the fragment centres will map upstream of the reference centre positions, whereas in libraries with predominantly non-overlapping start sites the centres will map upstream and downstream. We therefore used the data underlying the high-resolution overlap heatmaps to calculate the overlap start site ratio, which quantifies the number of fragment centres for each fragment length mapping to either the upstream or the downstream side of the reference centre position (see Method section for details). A ratio well above 1, such as the mean value of 1.31 for U2AF65, indicates that the use of the start positions of the iCLIP fragments will likely result in the most accurate binding site assignment. By contrast, a ratio below 1, such as the mean value of 0.88 for eIF4A3, favours the use of the centre position (Supplementary Tables 3 and 5). It is currently an open question how to determine the best mode of analysis when the ratio is close to 1, or where exactly to draw the threshold for the above-mentioned two clear-cut cases. In these cases it is useful to also consider the read distribution at particularly strong binding sites as shown in Fig. 3.

### Short and long reads show uridine enrichment at start sites

As a surprising finding from the read overlap heatmaps, we found a large number of fragments both in iCLIP and in CLIP libraries that overlapped at their end positions (Fig. 4 and Supplementary Fig. 4 bottom panels). We therefore compared the base composition around the fragment start sites and ends. For all iCLIP libraries analysed, we found an enrichment of thymidine (T) nucleotides around the start position when compared with the 10 nts upstream and downstream of the fragment start site (Fig. 5 and Supplementary Fig. 5). This is likely explained by the uridine preference of ultraviolet-C crosslinking and reflects the truncation in a sub-population of these iCLIP fragments at the crosslinking site^20^. We observed similar T enrichments around the start position of both short (group A) and long (group B) fragments, which indicates that both fragment types have similar truncation rates. As expected, we found this T enrichment to be particularly pronounced and to extend to upstream and downstream sequences around the start position in the iCLIP libraries of hnRNP L, U2AF65 and PTB, which are known to bind to T-rich motifs^15^,^16^,^18^,^22^ (Fig. 5c–f). In contrast, the PTB HITS-CLIP, but not iCLIP libraries, showed a sequence signature of RNase cleavage at fragment start sites, as is seen by the higher proportion of non-T-nucleotides at the fragment start sites (Fig. 5g and Supplementary Fig. 5g). Similar enrichment of non-T-nucleotides was found at the end sites of HITS-CLIP and iCLIP fragments with defined 3′ ends, suggesting that these also have a signature of RNase sequence preferences. In addition, these data show that the observed coinciding fragment ends are not caused by specific crosslinking events. One possible explanation for the alignment of fragment ends may therefore be the sequence preference of RNase, or that the RNA is protected by the RBP during the RNase digestion step of the iCLIP library preparation. Consistent with such protection from RNase digestion we observed, as explained above, a sharp drop in the frequency of fragment start sites at −30 nts and a sharp rise of fragment ends at −18 nts relative to the exon–exon junctions in the eIF4A3 iCLIP library (Fig. 3). This paucity of fragment start sites and fragment ends in this region (−30 to −18 nts) indicates a protection from RNase digestion and represents a footprint of eIF4A3 around the expected binding position 24 nts upstream of the exon–exon junctions.

Because the 3′ ends of completely sequenced fragments (group A fragments) signify the site of RNase cleavage, we restricted the input for the read overlap analysis to those fragments. We used the long fragments within group A, and not the incompletely sequenced group B fragments, as a reference. The outcome of this analysis is exemplified by the data obtained with eIF4A3 (Fig. 6) and matched the results using fragments of group B as a reference. We obtained similar results for hnRNP L, SRSF3, SRSF4 and PTB (Supplementary Fig. 6). These data indicate that the read overlap analysis is not significantly confounded by using the long fragments with undetermined 3′ ends (group B) as a reference (Supplementary Table 3). Therefore, both long and short fragments can be entered into the analysis thus increasing the coverage (Fig. 4 and Supplementary Fig. 4). Moreover, the analysis of our eIF4A3 and PTB iCLIP data sets showed that the use of the centre position of the fragments as a reference point aligned the mapping outcomes with previously published HITS-CLIP data sets of these proteins^12^,^18^, further supporting the choice of centre positions as reference points for the accurate mapping of binding site positions for iCLIP libraries with a majority of fragments that do not coincide in their start positions (Fig. 6 and Supplementary Figs 6 and 7).

## Discussion

As an alternative to HITS-CLIP^2^ and PAR-CLIP^4^,^5^, the development of iCLIP allows high precision analyses of RNA–protein interactions on a transcriptome-wide level^1^. Current analysis tools^6^,^21^,^23^,^24^,^25^ for iCLIP data sets are based on an assumption of narrow clustering of read start sites at sites of crosslinking, thus enabling the mapping of direct interaction sites of the RBP to the RNA with high precision (Fig. 7a). However, the first major finding reported here is that the proportion of fragments with coinciding fragment start sites within iCLIP data sets can differ. While some iCLIP libraries show the expected length-independent clustering of start sites, other libraries show predominantly non-overlapping start sites, whose mapping depends on the length of the iCLIP fragments, which leads to a definition of wide binding sites and can cause misassignment of the RBP interaction to a position upstream of the known binding site (exemplified for eIF4A3, Fig. 3 and Supplementary Fig. 7). However, it is important to note that the differences between libraries are quantitative and not absolute.

As a second contribution, we present analysis tools (high-resolution read distribution and overlap heatmaps) that improve the estimated positioning of protein–RNA interaction from iCLIP data. The high-resolution read overlap analysis (iCLIPro, http://www.biolab.si/iCLIPro/) visualizes and quantitates the overlap of start sites of iCLIP fragments of different lengths. The read overlap mapping has the advantage of being universally applicable to all RBPs and of being independent of the existence of an area of predominant binding. The read distribution mapping is useful for RBPs with known binding preferences such as consensus binding sites or distinct binding locations. For iCLIP libraries containing predominantly coinciding start sites, adjustments are not necessary and standard iCLIP analysis tools can be used to identify the binding sites. By contrast, for iCLIP libraries containing predominantly non-overlapping start sites, our new analysis tools improve binding site assignment. The key element of this assignment is the use of the centre positions of iCLIP fragments as the reference position, which allows the definition of narrower binding sites.

For iCLIP libraries containing predominantly non-overlapping start sites, we show that the use of the fragment centre as a reference point results in (1) a distribution with a narrower width compared with the use of the start of the fragment, (2) an improved overlap of the distributions generated by longer and shorter fragments and (3) assignment of the EJC binding sites to positions that are more consistent with the literature (Fig. 3 and Supplementary Fig. 7).

**Figure 1:**
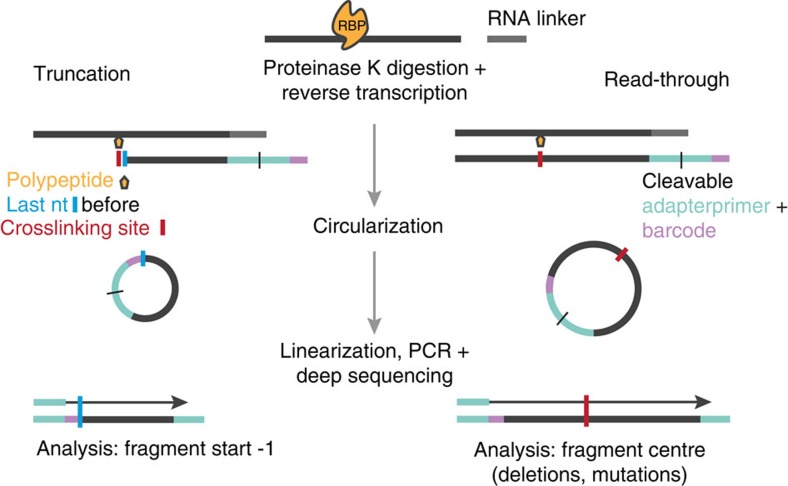
iCLIP generates fragments that result from either termination or read-through of the RT at the crosslinking site. Scheme of the iCLIP method (modified from ref. 6). The RNA fragment is covalently bound to the RNA-binding protein (RBP) after ultraviolet irradiation. After immunoprecipitation, the RBP is partially digested by proteinase K and, following RNA linker ligation, the RNA fragment is reverse transcribed. The RT either terminates or reads through at the crosslinking site (red bar) and thus iCLIP libraries can contain both truncated and full-length fragments. Following self-circularization, the complementary DNA is linearized, PCR amplified and deep-sequenced.

Which mechanism may explain these improvements? One plausible explanation posits that the RT bypasses the crosslinking site and predominantly reads through this position (Figs 1 and 7b top panel). This hypothesis is supported by the comparison of previously published HITS-CLIP data of eIF4A3 (ref. 12) with the iCLIP data of the same protein generated in our laboratory (Figs 3 and 6 and Supplementary Fig. 7).

Alternatively, the broadening of the distribution of fragment start sites in iCLIP libraries with non-overlapping start sites could be explained by multiple crosslinking sites of the same protein within a given region. This explanation may be particularly pertinent to proteins that (1) bind to long stretches of RNA sequence and (2) produce a strong footprint resulting in a high proportion of fragment ends to overlap (Fig. 7b bottom panel). For example, PTB contains four RNA recognition motif domains that can all crosslink to the RNA and thus could protect a long portion of its binding site from the RNase.

In principle, the differences in iCLIP patterns might also be caused by the chemistry of the binding of the RBP to the RNA. The eIF4A3 protein is known to bind to the RNA in a mostly sequence-independent manner^7^, although a low stringency sequence motif has recently been reported^11^,^12^. The interaction between the protein and the RNA might therefore occur at the phosphate backbone, whereas proteins with defined cognate binding motifs such as U2AF65 are likely to interact with the bases. However, because we found several proteins such as SRSF3, SRSF4 and PTB that recognize sequence motifs and yet generate iCLIP libraries with a large proportion of non-coinciding start sites, such a difference in binding chemistry does not offer a comprehensive explanation. We also considered whether the binding of an RBP to intronic versus exonic sequences could affect the proportion of coinciding fragment start sites in iCLIP libraries. However, the majority of fragment start sites in both, the predominantly exon-binding eIF4A3 and the intron-binding hnRNP L do not map to the same position in the reference genome. Another plausible explanation for non-overlapping start sites could be differences in RNA secondary structure. The deposition of RBPs to the RNA can be hindered by local secondary structures^11^,^26^,^27^. Recently developed techniques without ultraviolet crosslinking like RNA Bind-n-Seq (RBNS)^27^ are tailored to identify different structural binding specificities of RBPs and can be used to complement and strengthen the CLIP results.

Whether a single mechanism is responsible for the differences between the iCLIP data sets remains an open question. However, regardless of the underlying mechanism(s), the overlap analysis of the start sites of iCLIP fragments of different lengths can be used as a diagnostic tool to detect data sets that would be misinterpreted by current standard approaches. Importantly, the use of the fragment centres will assign narrower binding sites in iCLIP libraries that contain a high proportion of non-coinciding fragment start sites. Alternatively, deletions or mutations of the sequenced fragments, which the RT may introduce at the crosslinking site in case read-through occurs, can also be exploited for high-resolution mapping of individual binding sites^28^,^29^,^30^,^31^. However, such an approach requires a high frequency of such deletions and mutations, which have been shown to vary widely between different RPBs^20^,^21^ (see also Supplementary Table 1) and can thus not be used in all cases. In addition, a recent study by Weyn-Vanhentenryck *et al.*^21^ directly compared HITS-CLIP and a modified iCLIP (BrdU-CLIP) approach and showed that the estimated truncation rates can be variable for different RBPs. Although we show that, in cases of predominantly non-coinciding start sites, the use of the centre can improve the detection and assignment of binding sites compared with standard iCLIP tools (Supplementary Fig. 7), the use of the centre may not always result in high-resolution assignment of the binding site. However, the identification of non-coinciding start sites in iCLIP libraries raises awareness of potential binding site misassignments and testing the centre position can serve as a simple and pragmatic solution to improve the iCLIP analysis. Because the use of the centre will not provide single nucleotide resolution, novel biochemical and bioinformatical approaches for CLIP technologies will have to be developed. Moreover, the identification of mechanisms underlying the occurrence of non-coinciding fragment start sites for specific RBPs will help improving the quality of iCLIP libraries and binding site assignment. Hence, the performance of CLIP experiments for diverse RBPs and the development of methods that directly measure the truncation rate are necessary to deepen the understanding of protein–RNA interactions.

The similar outcome of the high-resolution read overlap heatmap analyses against a reference of (1) fragments with undetermined 3′ ends (group B, Fig. 4 and Supplementary Fig. 4) and (2) completely sequenced fragments (group A, Fig. 6 and Supplementary Fig. 6) indicate that the results are not significantly confounded by the read length. However, to obtain high-resolution binding profiles in practice we recommend optimizing the iCLIP experiment to cover a wide range of fragments with different length, because the resolution of the read distribution and overlap heatmaps depend on the number of fragments with different length. This can be best achieved by thoroughly controlling the biochemical part of iCLIP protocols, for example, by optimizing the RNase treatment^32^ and by increasing the read length to yield a larger proportion of fragments that are completely sequenced. This selection enables the identification of a distribution of the centres and ends of fragments, and allows to determine and to score the proportion of fragments with overlapping start sites of the resulting libraries.

In summary, we identify a previously unrecognized effect of iCLIP fragment length on the position of fragment start sites and thus assigned binding sites for some RBPs, and present a robust analysis approach that examines this effect to improve the assignment of binding sites from iCLIP data.

## Methods

### Cell culture and iCLIP

HeLa cells were cultured in DMEM, supplemented with 10% (v/v) FCS and penicillin/streptomycin under 5% CO_2_ at 37 °C. For iCLIP, HeLa cells expressing eIF4A3–green fluorescent protein (GFP) or PTB–GFP were induced with doxycycline to adjust the level of recombinant protein to the level of the endogenous counterpart and irradiated with 150 mJ cm^−2^ ultraviolet light (254 nm). After lysis, recombinant proteins were immunoprecipitated with 30 μl GFP–Trap_A (Chromotek, catalogue number: gta-20), RNase I-treated (final concentration 0.02 U μl^−1^) and subsequent iCLIP steps were performed as described^6^.

### HITS and preprocessing of reads

The iCLIP libraries for eIF4A3 and PTB were sequenced with 50 nts run length on an Illumina HiSeq 2,000 instrument. For the other iCLIP libraries, FASTQ files were downloaded from the corresponding publication sources (Table 1).

The Galaxy environment was used for the post-processing of the reads^33^,^34^,^35^. Briefly, Jemultiplexer (version 1.0.0) from the Galaxy environment was used to demultiplex the different experiments by their sample barcodes. The sample barcode together with the random barcode were removed from the reads and stored in the read header for later evaluation and removal of duplicates.

After removing the barcode, the end of the iCLIP reads were analysed for the presence of a potential 3′ Solexa primer using the FASTX-Toolkit. The reads containing parts of the 3′ Solexa primer were trimmed from the 3′ end.

### Analysis of fragment length

**Figure 2:**
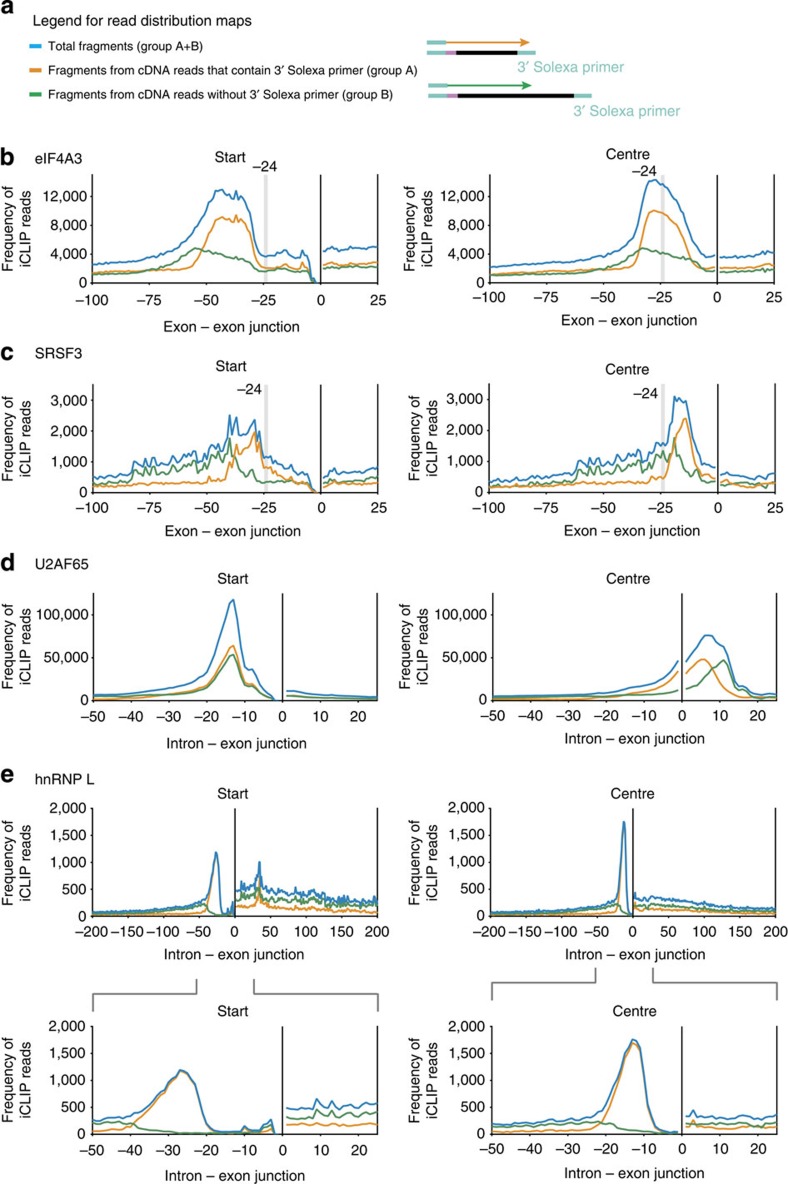
Read distribution maps for several RNA-binding proteins. (**a**) The entire population of random barcode evaluated fragments was subdivided into fragments that were either sequenced completely and hence the corresponding sequence read (orange arrow) contained parts of the 3′ Solexa primer (group A) or fragments that were longer than the sequence reads and the corresponding read (green arrow) hence did not contain parts of the 3′ Solexa primer (group B). The graphs of (**b**) eIF4A3, (**c**) SRSF3, (**d**) U2AF65 and (**e**) hnRNP L show the distributions of the entire population of fragments (blue), fragments of group A (orange) and group B (green). The left and right columns show the mapping of, respectively, the start and the centre positions of the iCLIP fragments. The fragment lengths (different for each RBP) used for the assignment to these sub-groups are given in Supplementary Table 2.

To analyse the impact of the fragment length, reads were separated into reads that either did or did not contain parts of the 3′ Solexa primer (group A and group B, respectively; Fig. 2a). This separation allowed for the differentiation between shorter fragments with known and variable fragment sizes (group A) from fragments with a length equal or longer than the read length (group B).

### Mapping to the reference genome

Group A, group B and total fragments were mapped to the reference genome (assembly GRCh37, as provided by Ensembl 73 for human; and assembly GRCm38, as provided by Ensembl 74 for mouse data) using STAR version 2.3.0 (ref. 10). Prior to mapping, an index containing an exon–exon junction database was generated with an overhang of 43 nts for the human and 46 nts for the mouse reference genome by STAR. A total mismatch rate of 2 was allowed for mapping (--outFilterMismatchNmax 3, --outFilterMismatchNoverLmax 0.12). For the other parameters default settings were used. Using different mismatch thresholds (0 to 10) or another mapping programme (default parameters of TopHat 2 together with Bowtie 2)^36^ did not affect the overall outcome of this analysis (Supplementary Fig. 8).

### Generation of read distribution maps

**Figure 3:**
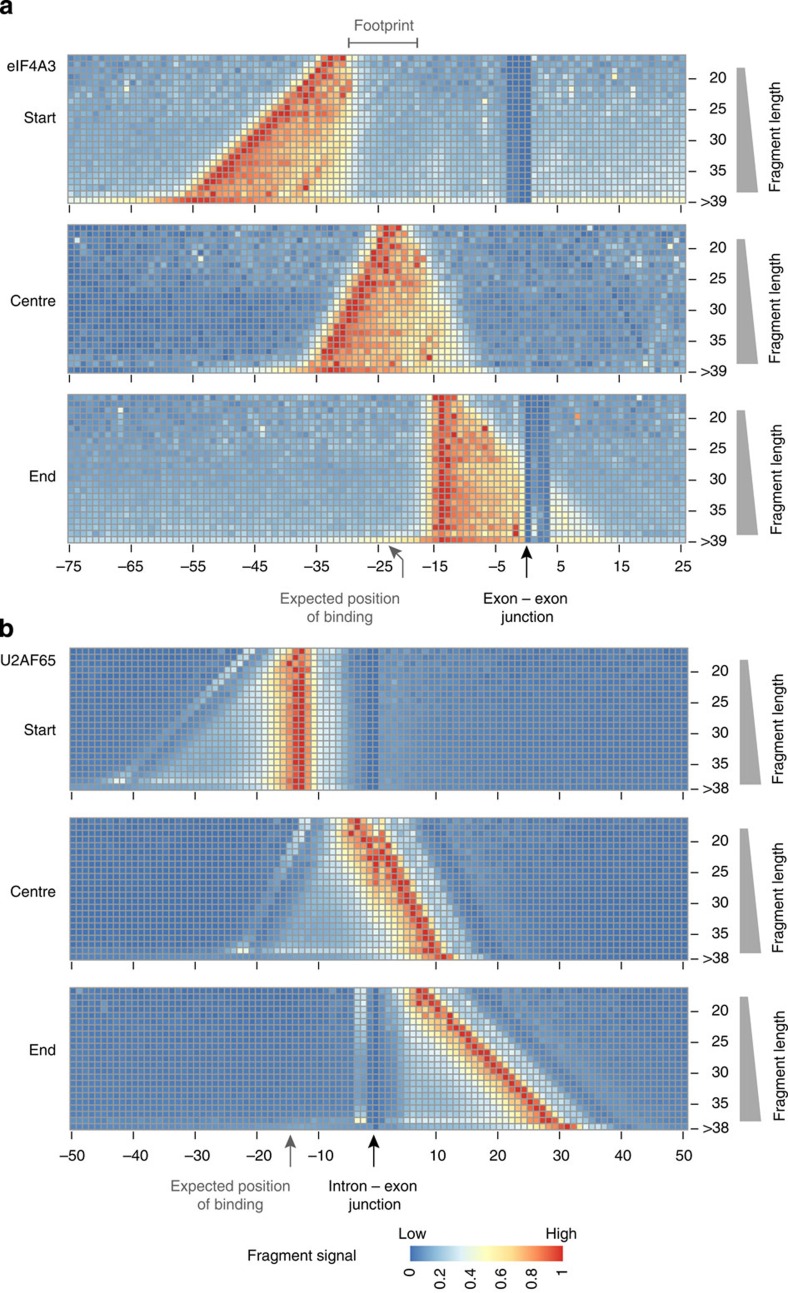
High-resolution read distribution heatmaps for eIF4A3 and U2AF65. Each row shows the histogram of the fragment start (top panel), centre (middle panel) and end (bottom panel) positions, stratified by fragment length as indicated on the *y*-axis, and with histogram bin heights represented by colours. The positions are plotted relative to (**a**) the exon–exon junction for eIF4A3 and (**b**) the intron–exon junction for U2AF56. The distributions are normalized by the number of fragments of the respective length. The number of fragments for each length is shown in Supplementary Fig. 1.

After mapping to the reference, duplicates were removed by using the random barcodes for each read with a custom Python script. For each iCLIP experiment, the library with the most unique hits to the reference genome was used to generate the read distribution as well as the read overlap maps. The mapped reads with an alignment quality score of ≥10 were used to generate read distribution maps of the ends, the centres and the nucleotide preceding the starts of the fragments (Figs 2 and 3). In the case of the subset of reads where no 3′ Solexa primer was detected, the centre and end of the read was used for the analysis.

The read distribution maps were drawn from the distances of the fragment ends, centres or the nucleotide preceding the fragment starts to the beginning and the end of annotated exons or introns (GRCh37.73 or GRCm38.74). The distances were calculated using a custom script based on the Python framework HTSeq^37^. The high-resolution read distribution heatmaps were normalized by the number of fragments per each fragment length in the analysed window size.

### Generation of high-resolution read overlap heatmaps

**Figure 4:**
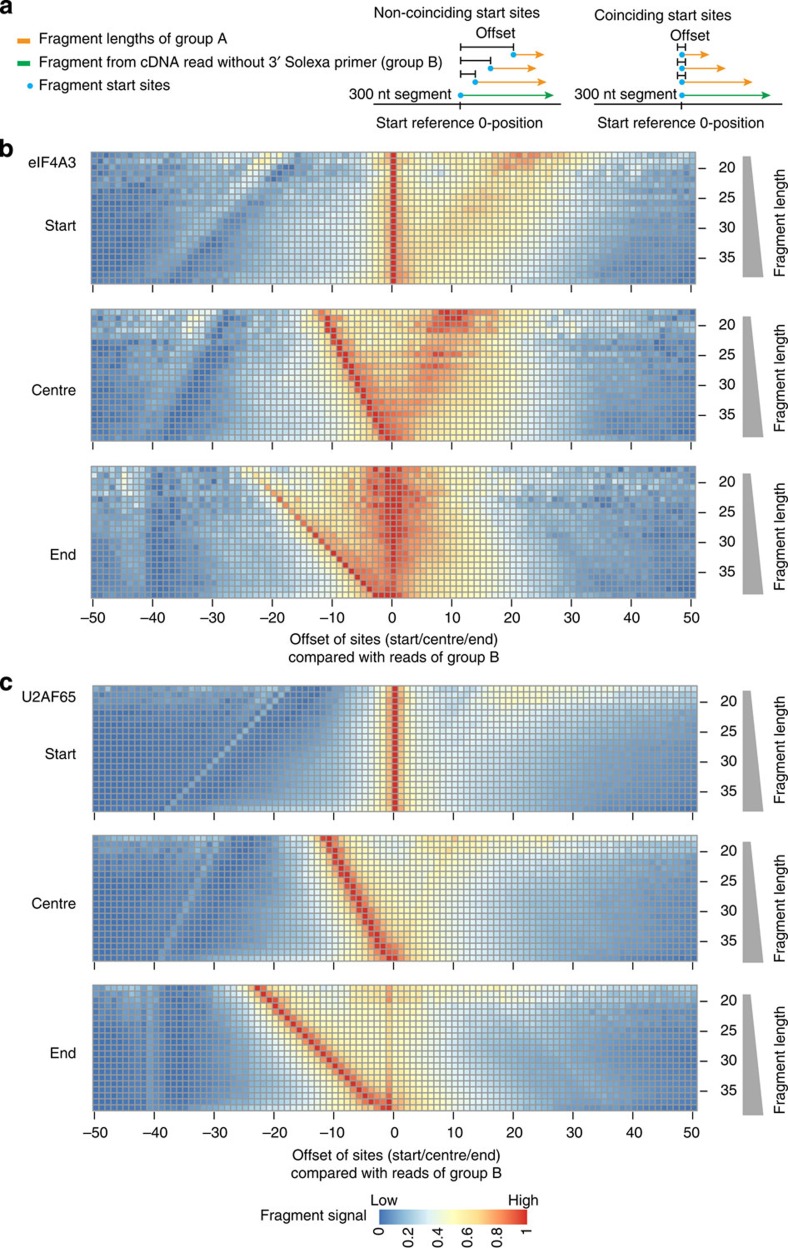
High-resolution read overlap heatmaps for the start, centre and end positions of iCLIP fragments for eIF4A3 and U2AF65. (**a**) Schematic representation of the method. The high-resolution read overlap heatmaps are shown for (**b**) eIF4A3 (mean overlap start site ratio 0.88) and (**c**) U2AF65 (1.31). For this analysis, the distance of the start sites between random barcodes evaluated fragments that map to the same region at the reference genome is plotted on the *x*-axis (**b** and **c** top panel). As a reference, the start sites of the group B fragments are used to calculate the distance to the start site of the fragments of group A. A peak at the reference position 0 corresponds to coinciding start sites of group B and group A fragments, whereas a distribution downstream of the reference position 0 arises from the start sites of group A fragments that occur at length-dependent offsets from the group B start sites. The same procedure was applied to the centre (**b** and **c** middle panel) and end positions (**b** and **c** bottom panel) of the sequenced fragments (group A and B). The number of fragments for each fragment length is shown in Supplementary Fig. 1.

As for the read distribution maps, the fragments were split into groups by their length. For the high-resolution read overlap heatmaps, we subdivided the genome into segments of 300 bp and focused on those segments that contain at least 50 iCLIP reads (20 iCLIP reads for low coverage libraries SRSF4, TIAL1 and PTB Ule lab, Fig. 4 and Supplementary Fig. 4). In the software, the segment size and read number parameters can be adjusted according to the coverage of iCLIP fragments and the needs of the user (iCLIPro, http://www.biolab.si/iCLIPro/). Within each segment, we defined the start (centre or end) positions of the reference fragments as the 0-positions. For each reference set of positions separately, the regions (−50 to +50, *x*-axis) relative to the reference positions were then scanned and the numbers of co-occurring fragments was recorded. The *x*-axis shows the offset of the shorter fragment relative to the position of the longer fragments. The *y*-axis shows the fragment length. The colour in the heatmap represents the number of fragments that co-occur at a given offset relative to the longer reference fragments. The procedure was repeated for the second and third set of high-resolution read overlap maps, with the only difference that the centre and end of fragments were used to identify the fragment positions.

### Calculation of the start site overlap ratio

We used the data underlying the high-resolution overlap heatmaps to calculate a ratio of the number of fragment centres mapping either upstream or downstream of the reference centre position as defined above. The centres of fragments with coinciding start sites will be mapped upstream of the reference position creating a length-dependent slope corresponding to the fragment length-specific maximal offset between the centre and the reference positions. We determined the number of fragments within the range of the reference 0 position and this maximal offset (+5 nts flanking region) upstream and downstream of the reference 0 position for each fragment length. We then divided the mean of fragment centres upstream by the mean of the fragment centres downstream of the reference position within this range for each fragment length (start site overlap ratio per fragment length). The iCLIPro software finally reports the mean and the median of these individual start site overlap ratios. A ratio >1 indicates that the use of the start positions of the iCLIP fragments will result in most accurate binding site assignment, whereas a ratio <1 favours the use of the centre position.

### Nucleotide composition

**Figure 5:**
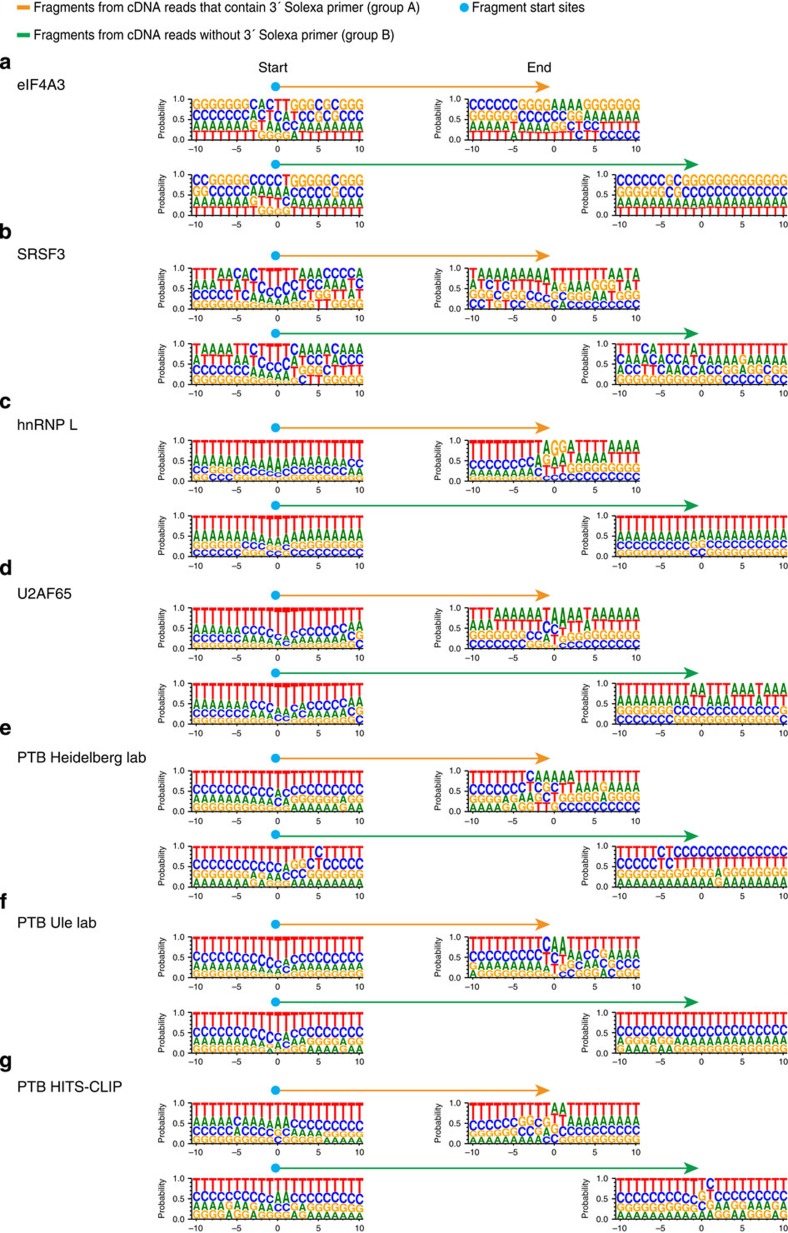
Nucleotide compositions around start and end sites of CLIP fragments. Fragments were divided into separate categories as described in Fig. 2a, and nucleotide composition was examined around start and end of fragments of different lengths at the predominant binding sites for iCLIP libraries of (**a**) eIF4A3, (**b**) SRSF3, (**c**) hnRNP L, (**d**) U2AF65, (**e**) PTB Heidelberg lab, (**f**) PTB Ule lab and (**g**) a HITS-CLIP library of PTB.

**Figure 6:**
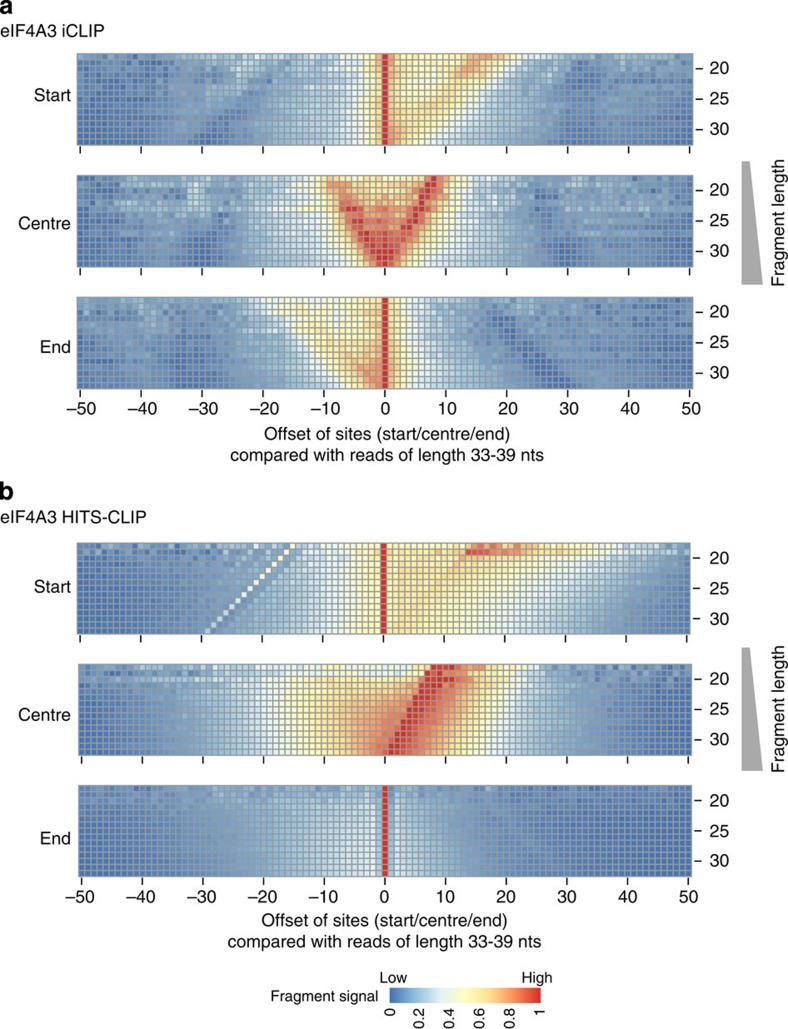
High-resolution read overlap heatmaps for different positions of fragments from an iCLIP and a HITS-CLIP library for eIF4A3. (**a**) Analysis of the iCLIP library generated in our laboratory (mean overlap start site ratio 0.97). (**b**) Re-analysis of previously published data obtained with HITS-CLIP^12^ (0.81). The read overlap maps were generated as described in the legend of Fig. 4a, with the difference that completely sequenced fragments of group A with a length of 33–39 nts are used for defining the reference 0-positions of either the start site (top panel), the centre (middle panel) and the end (bottom panel) of the fragments. In HITS-CLIP, only read-through occurs and thus the centre positions of the fragments are statistically distributed around the crosslinking site. The distribution of the fragment lengths are given in Supplementary Fig. 1.

**Figure 7:**
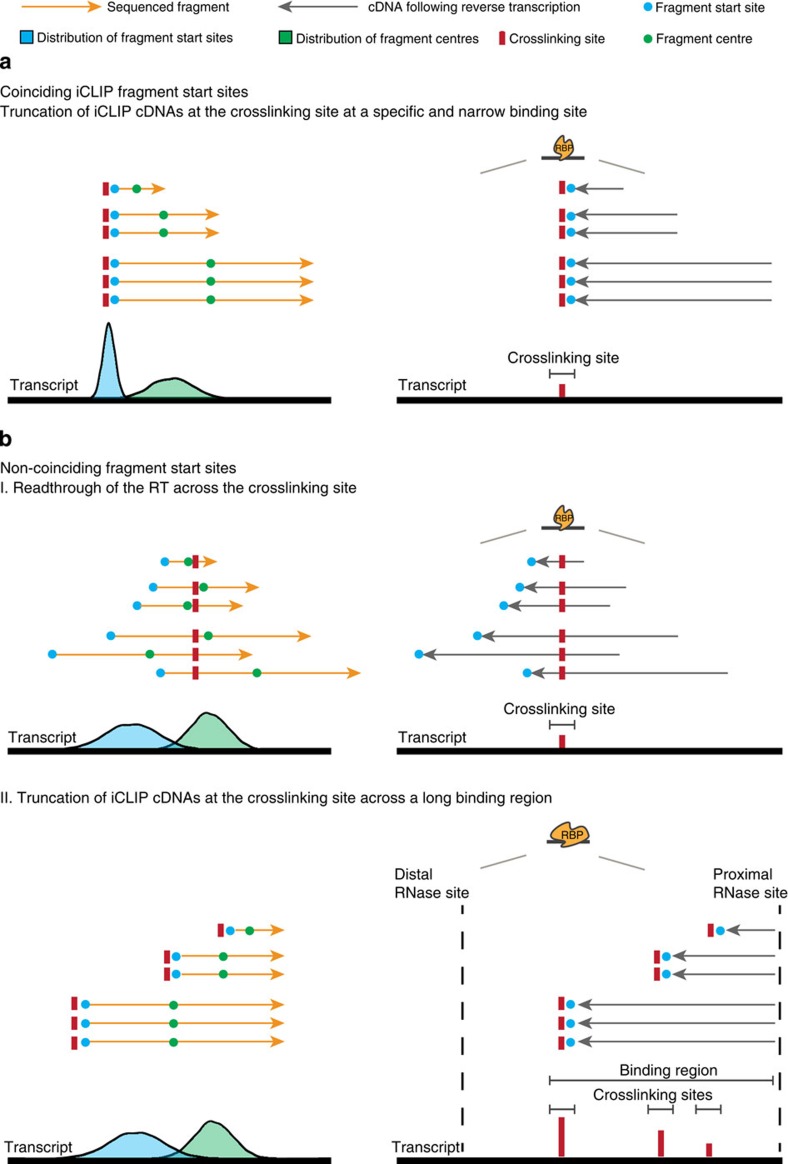
Schematic representations of potential mechanisms at the reverse transcription step in iCLIP. (**a**) Coinciding iCLIP fragment start sites. The crosslinking site can be defined in a manner independent of fragment length as the nucleotide preceding the iCLIP fragment, if RT synthesis is dominated by truncation. The distribution of other reference points within the iCLIP fragment such as the centre will be much broader when compared with the distribution of the start sites, because of the different lengths of the sequenced fragments. (**b**) Non-coinciding iCLIP fragment start sites. (I) A read-through mechanism or (II) long crosslinking regions are plausible explanations for a broad distribution of iCLIP fragment start sites. In case of read-through, the centre positions of the iCLIP fragments will be closer to the distribution of the crosslinking sites than the start sites of the iCLIP fragments. In case the protein binds and crosslinks across a long stretch of RNA, the iCLIP fragments of different lengths may sample different crosslinking sites. The overlap of iCLIP fragment ends suggests that the protein protects the RNA from RNase digestion. In case of predominantly non-overlapping start sites the use of the fragment start site as a reference point for mapping binding sites is dependent on the fragment length distribution (see Supplementary Fig. 1). The use of the centre position will reduce this length-dependency and improves the assignment of binding sites due to the increased signal of overlapping centres compared with start sites of the fragments.

iCLIP reads were divided into separate categories as described in Fig. 2a and Supplementary Table 2. The nucleotide composition was examined around start and end of fragments of different lengths at the predominant binding sites (1–100 nt upstream of exon–exon or intron–exon junction) using the weblogo 3.3 software^38^ (Fig. 5 and Supplementary Fig. 5).

### Cluster of crosslinking sites

We used the iCount default analysis to detect and cluster crosslinking sites (iCount, http://icount.biolab.si) as described previously^6^,^20^. To validate our findings, we performed the crosslinking induced truncation sites method with the recommended default parameters (*P*<0.001 and cluster sites within a window of 25 nts)^21^. In addition to the analysis of the nucleotide preceding the start sites of the fragments, we used the centre of iCLIP fragments as an input.

## Additional information

**Accession codes:** FASTQ files of eIF4A3 and PTB iCLIP libraries can be downloaded from ArrayExpress with accession code E-MTAB-2599.

**How to cite this article:** Hauer, C. *et al.* Improved binding site assignment by high-resolution mapping of RNA–protein interactions using iCLIP. *Nat. Commun.* 6:7921 doi: 10.1038/ncomms8921 (2015).

## Supplementary Material

Supplementary Information Supplementary Figures 1-8, Supplementary Tables 1-5 and Supplementary References

## Figures and Tables

**Table 1:** Characteristics of the RNA-binding proteins used.

	**Predominant binding region**	**iCLIP or HITS-CLIP**	**Cells**	**Identifier of raw sequences**
**eIF4A3**	Upstream of exon junction (exonic)	iCLIP	HeLa	E-MTAB-2599
		HITS-CLIP	HeLa	SRR567526.1 (ref. 12)
**SRSF3**	Upstream of exon junction (exonic)	iCLIP	P19 (mouse)	ERR039837 (ref. 13)
**SRSF4**	Upstream of exon junction (exonic)	iCLIP	P19 (mouse)	ERR039839 (ref. 13)
**PTB**	3′ splice site (intronic)	iCLIP Heidelberg lab	HeLa	E-MTAB-2599
		iCLIP Ule lab	HeLa	E-MTAB-3108 (ref. 17)
		HITS-CLIP	HeLa	SRR034466 (ref. 18)
**U2AF65**	3′ splice site (intronic)	iCLIP	HeLa	ERR196183 and ERR196190 (ref. 15)
**hnRNP L**	3′ splice site (intronic)	iCLIP	HeLa	SRX144295 (ref. 16)
**TIAL1**	5′ splice site (intronic)	iCLIP	HeLa	E-MTAB-526 (ref. 14)

HITS, high-throughput sequencing; iCLIP, individual-nucleotide resolution crosslinking and immunoprecipitation.
