# Supplementary material for: Improved binding site assignment by high-resolution mapping of RNA–protein interactions using iCLIP
Source: Nat Commun. 2015 Aug 11;6:7921. doi: 10.1038/ncomms8921 (PMC4918375; doi:10.1038/ncomms8921)
Supplement: Supplementary Information — Supplementary Figures 1-8, Supplementary Tables 1-5 and Supplementary References [file ncomms8921-s1.pdf]

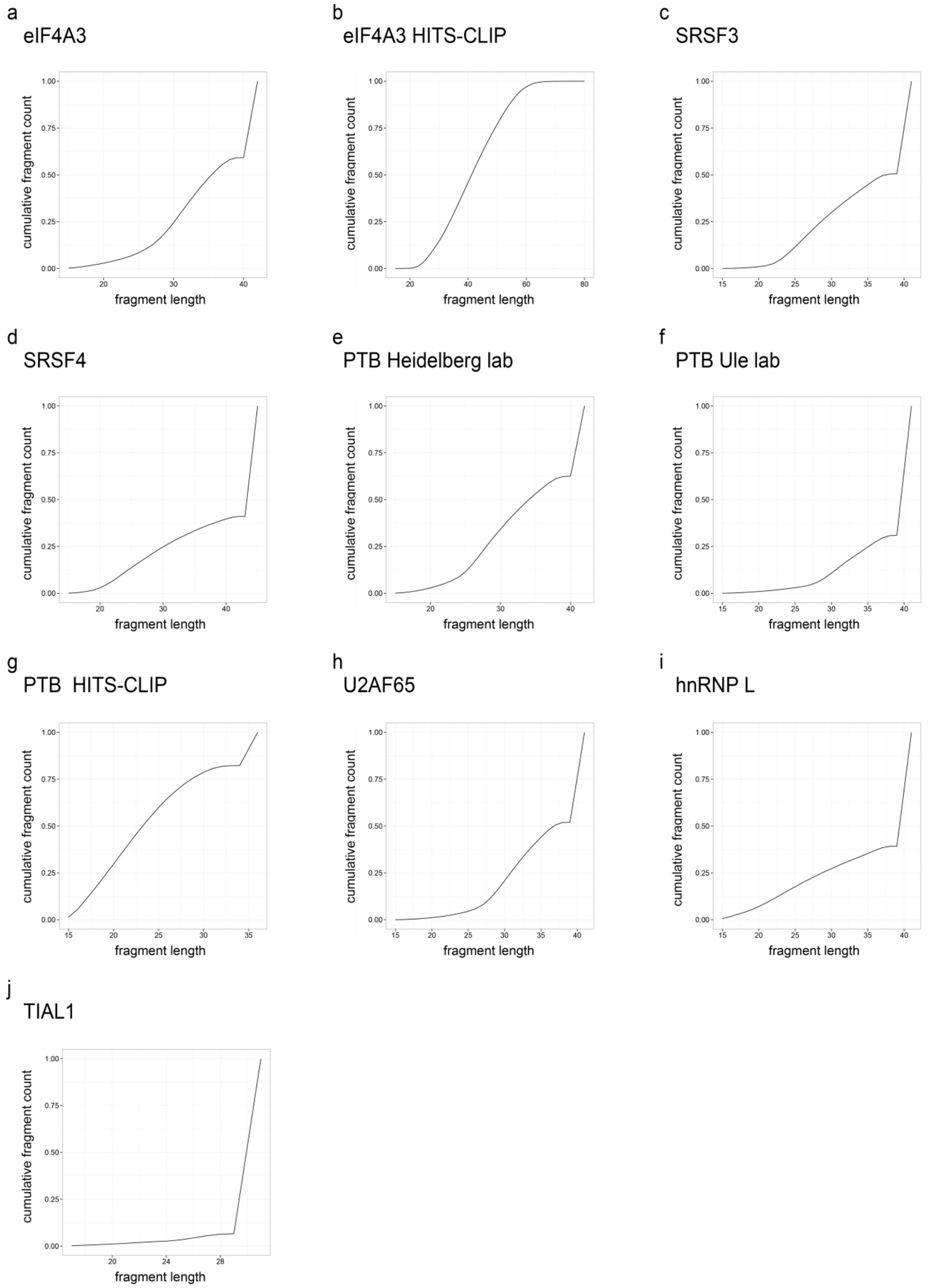

Supplementary Figure 1 Fragment length distributions of (**a**) eIF4A3, (**b**) eIF4A3 HITS-CLIP, (**c**) SRSF3, (**d**) SRSF4, (**e**) PTB Heidelberg lab, (**f**) PTB Ule lab, (**g**) PTB HITS-CLIP, (**h**) U2AF65, (**i**) hnRNP L, and (**j**) TIAL1. The sharp increase of fragments corresponds to fragments of group B (see **Supplementary Table 2**).

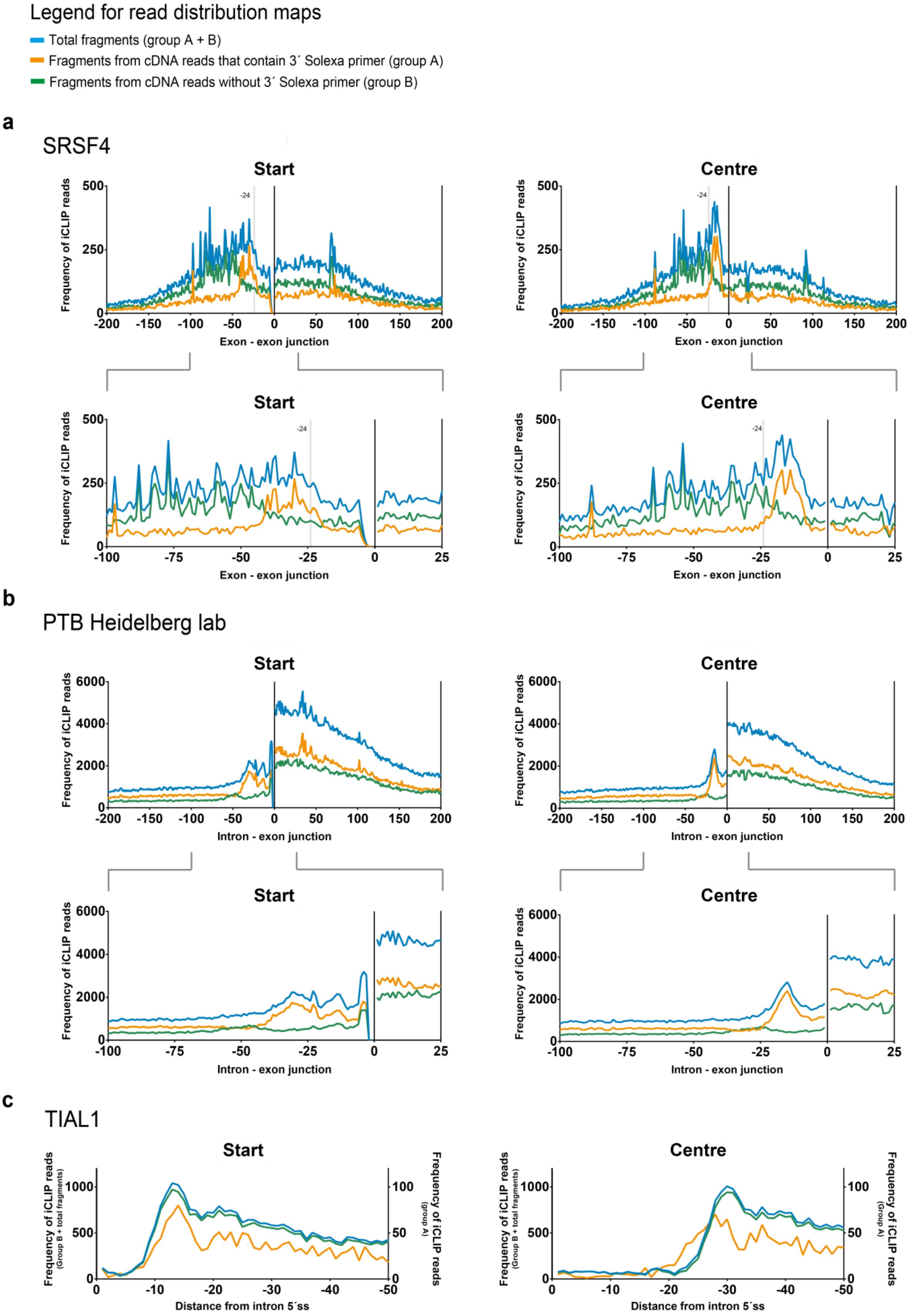
Legend for read distribution maps
Total fragments (group A + B)
Fragments from cDNA reads that contain 3´ Solexa primer (group A)
Fragments from cDNA reads without 3´ Solexa primer (group B)
a
SRSF4
Start
Centre
Frequency of iCLIP reads
-24
Exon - exon junction
b
PTB Heidelberg lab
Start
Centre
Frequency of iCLIP reads
Intron - exon junction
c
TIAL1
Start
Centre
Frequency of iCLIP reads (Group B + total fragments)
Frequency of iCLIP reads (group A)
Distance from intron 5´ss

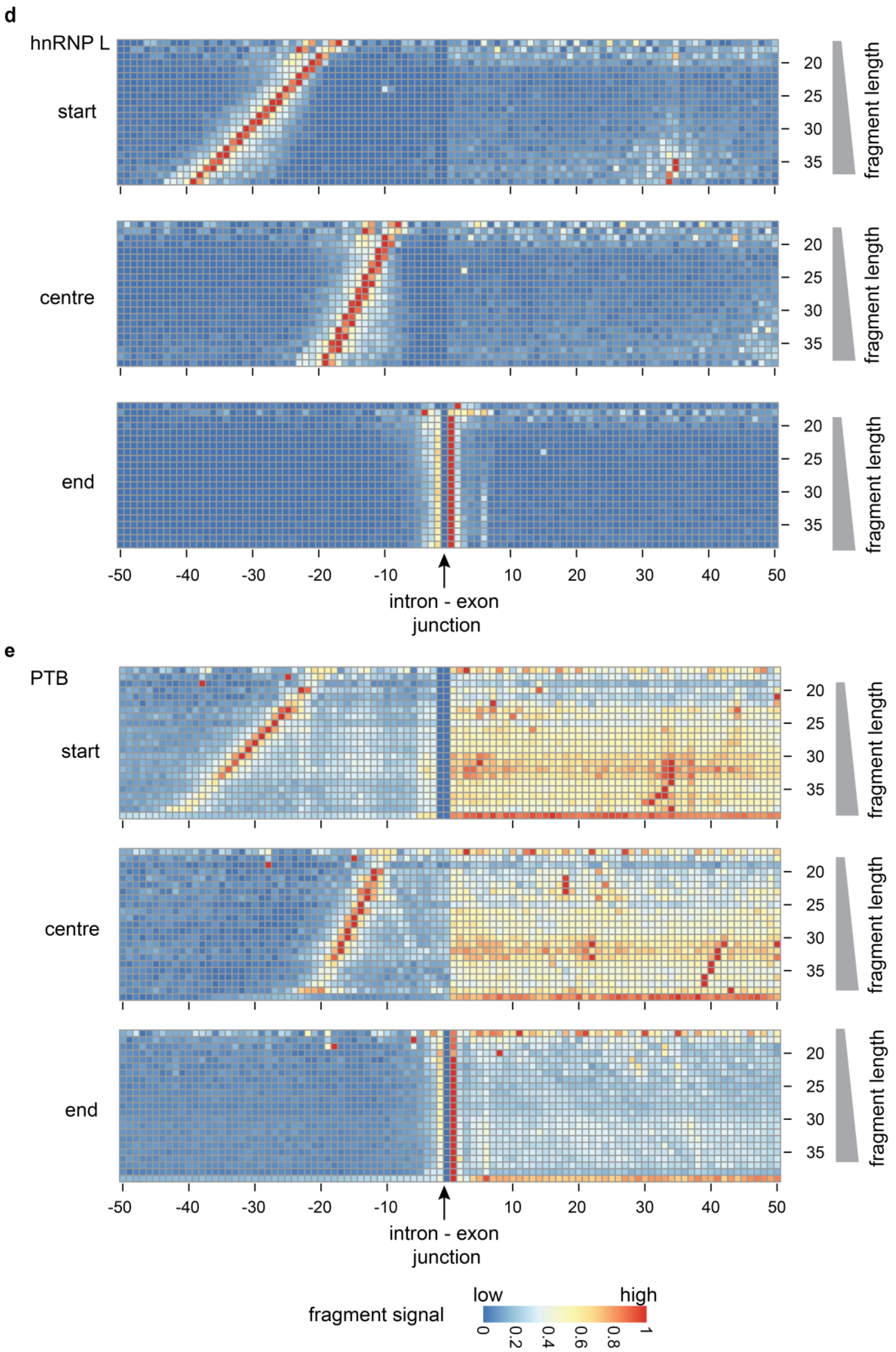

Supplementary Figure 2 Read distribution maps for (**a**) SRSF4, (**b**) PTB Heidelberg lab, (**c**) TIAL1, and high-resolution read distribution heatmaps for (**d**) hnRNP L and (**e**) PTB Heidelberg lab. See legend of **Figure 2** and **3** for explanations. Abbreviations: 5´ss = 5´splice site.

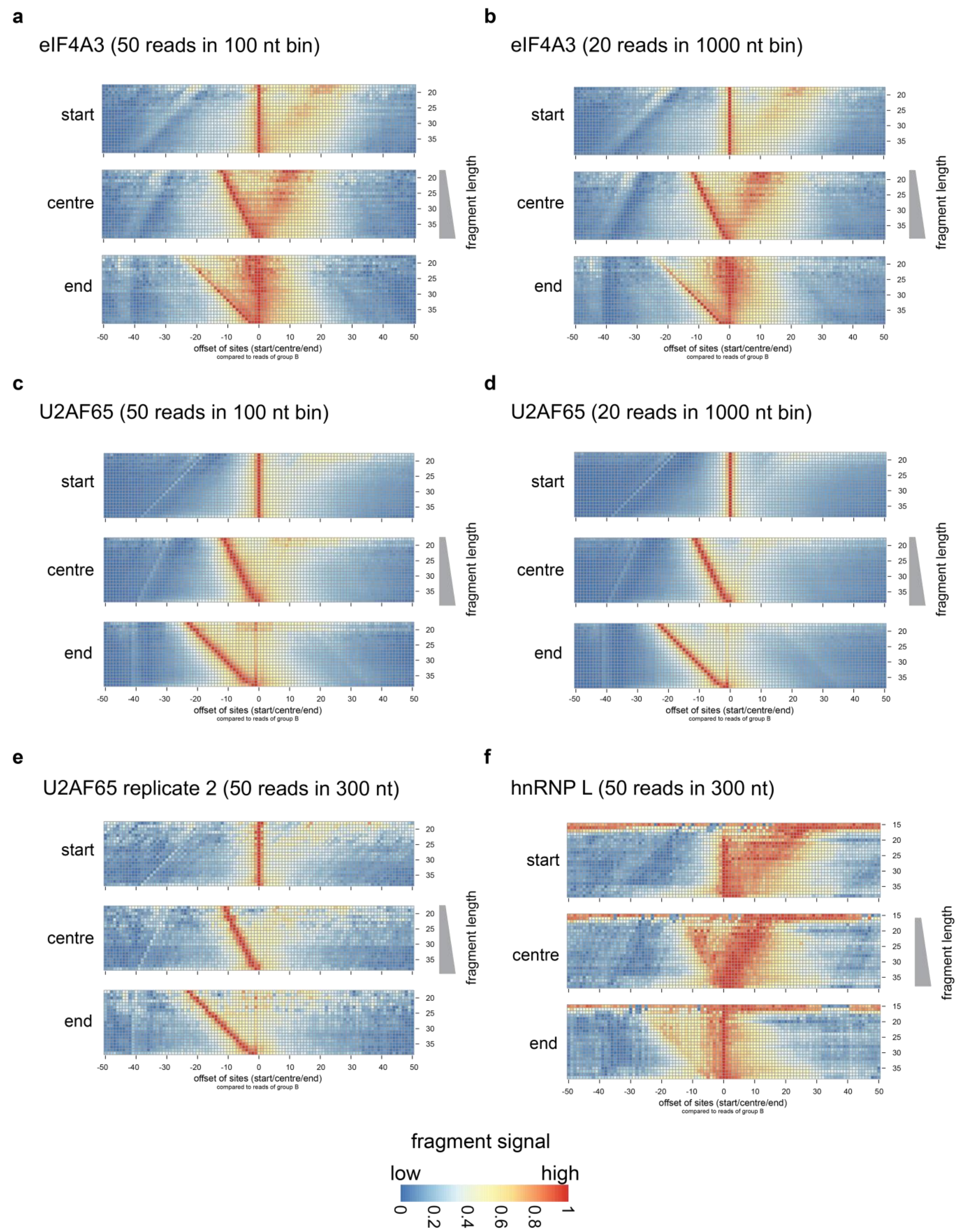

Supplementary Figure 3 High-resolution read overlap heatmaps of iCLIP fragments to test parameters. (**a-d**) High-resolution read overlap heatmaps of eIF4A3 and U2AF65 using different bin sizes and number of reads per bin were generated to test the robustness of the tool. The mean overlap start site ratios are (**a**) 0.90, (**b**) 0.86, (**c**) 1.27, (**d**) 1.41. (**e**) High-resolution read overlap heatmaps of U2AF65 replicate 2 to test the influence of the library size. The replicate 2 consists of less reads

(1,380,687 reads, ERR196190, see **Supplementary Table 1** for other libraries) compared to replicate 1 (8,958,729 reads) after duplicate removal and quality filtering. The distributions and mean overlap start site ratios (replicate 2: 1.32; replicate 1: 1.31, see **Fig. 4**) are highly similar between the two replicates. (**f**) High-resolution read overlap heatmaps of hnRNPL (mean overlap start site ratio 0.76). The short reads below 18 nts give rise to strong noise that has to be excluded from the calculation (for read coverage see **Supplementary Fig. 1**). In **Supplementary Figure 4** the high-resolution read overlap heatmaps are depicted without the short reads with low coverage for the respective datasets.

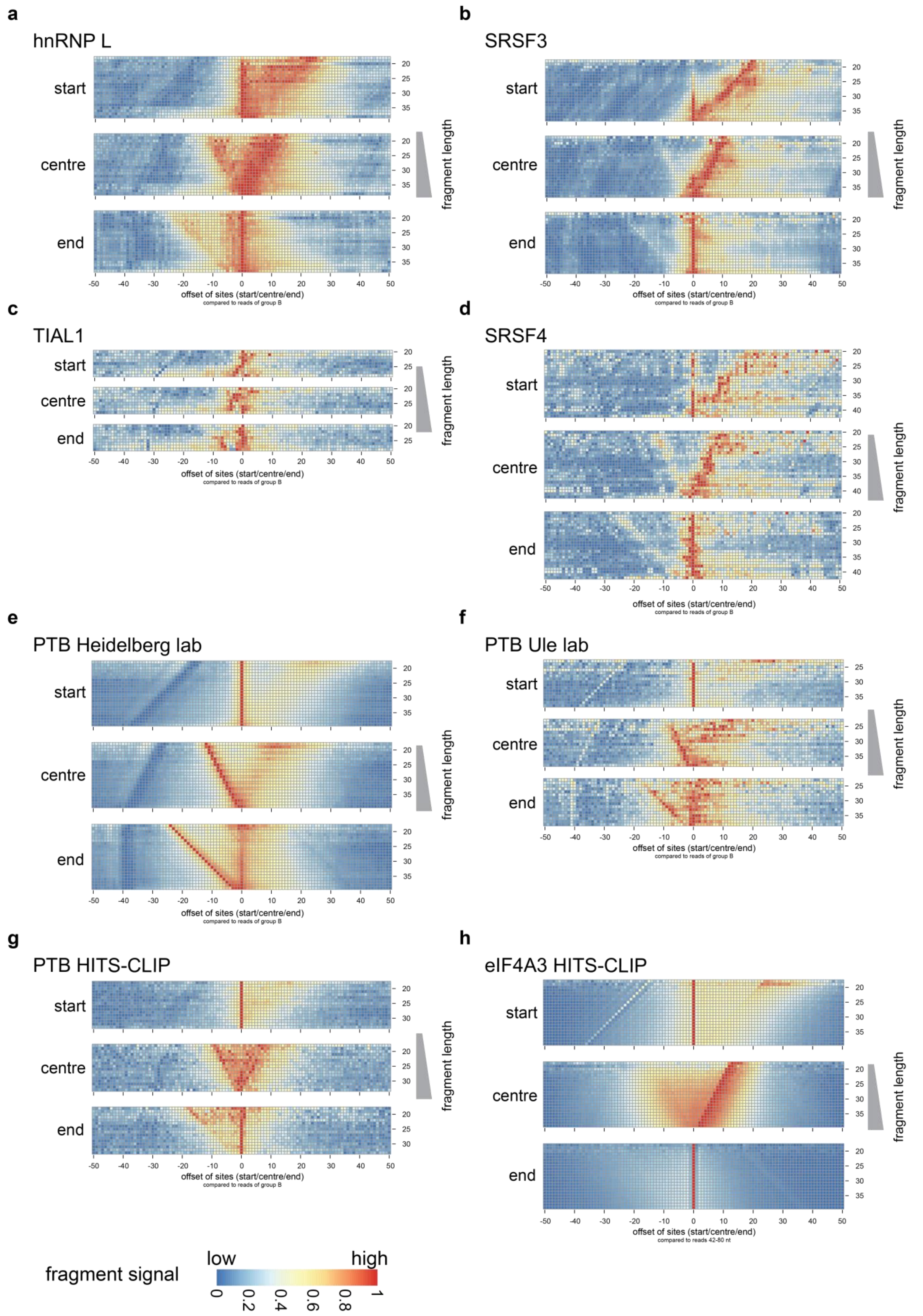

Supplementary Figure 4 High-resolution read overlap heatmaps for the start, centre and end positions of iCLIP fragments. The high-resolution read overlap heatmaps for the different RBPs are shown for (**a**) hnRNP L (mean overlap start site ratio 0.77), (**b**) SRSF3 (0.43), (**c**) TIAL1 (1.04), (**d**)

SRSF4 (0.50), (**e**) PTB Heidelberg lab (0.92), (**f**) PTB Ule lab (0.89), (**g**) PTB HITS-CLIP (0.95), and (**h**) eIF4A3 HITS-CLIP (0.83). The high-resolution read overlap heatmaps were generated as described in **Figure 4**.

a

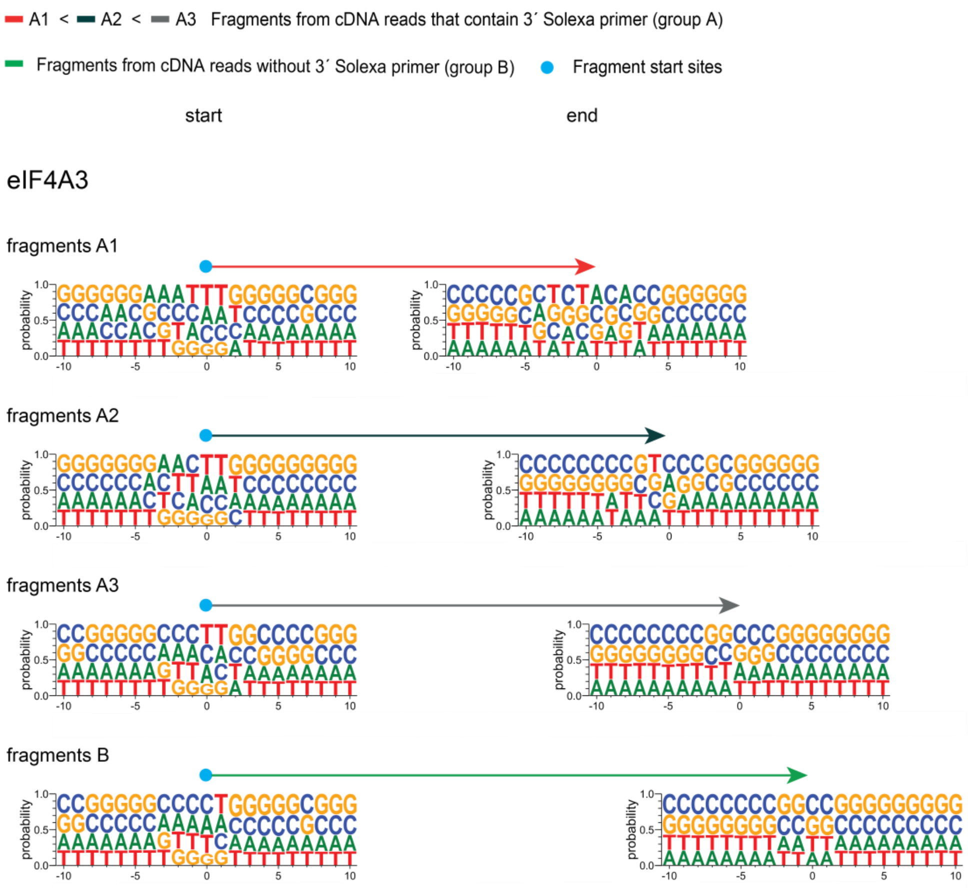

A1 < A2 < A3 Fragments from cDNA reads that contain 3´ Solexa primer (group A)
Fragments from cDNA reads without 3´ Solexa primer (group B)
Fragment start sites
start
end
eIF4A3
fragments A1
fragments A2
fragments A3
fragments B
probability

b

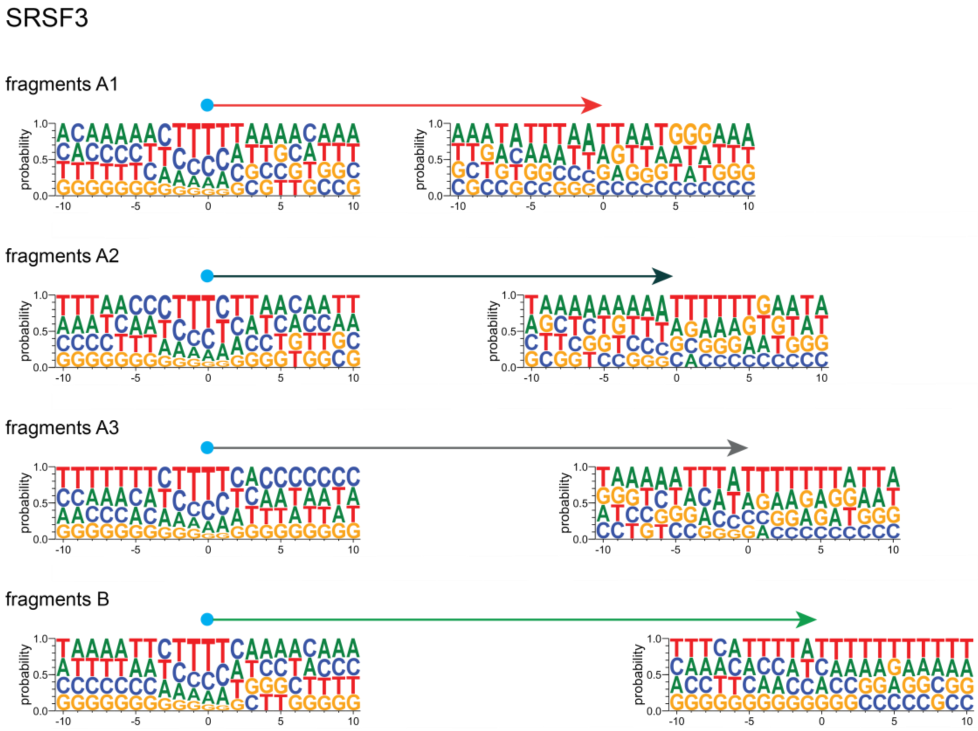

SRSF3
fragments A1
fragments A2
fragments A3
fragments B
probability

c

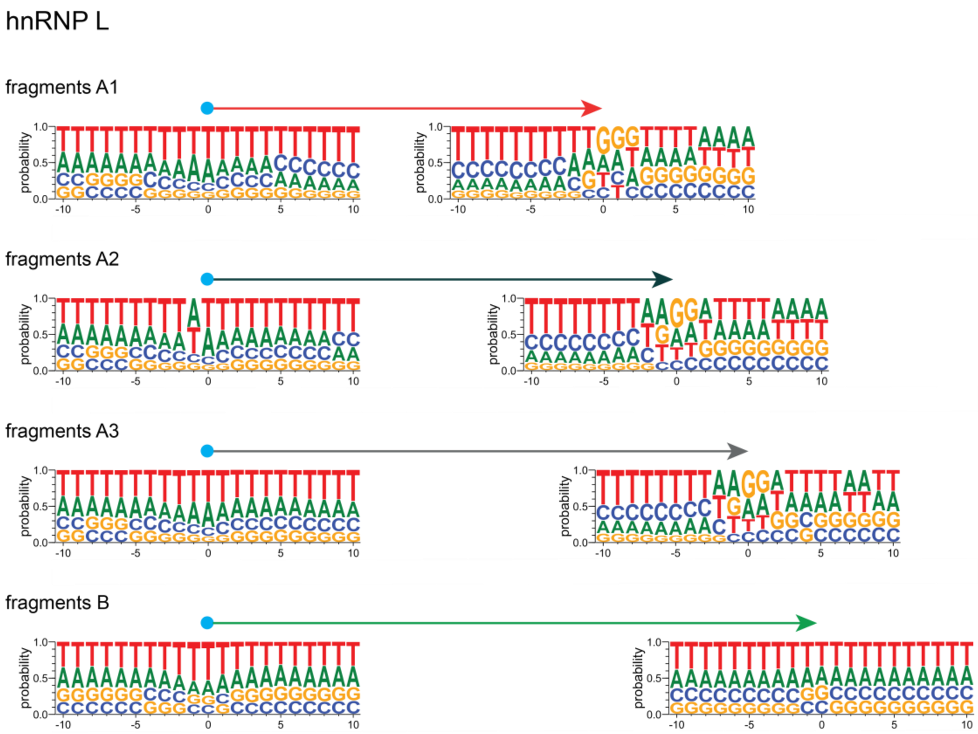
hnRNP L
fragments A1
fragments A2
fragments A3
fragments B
probability

d

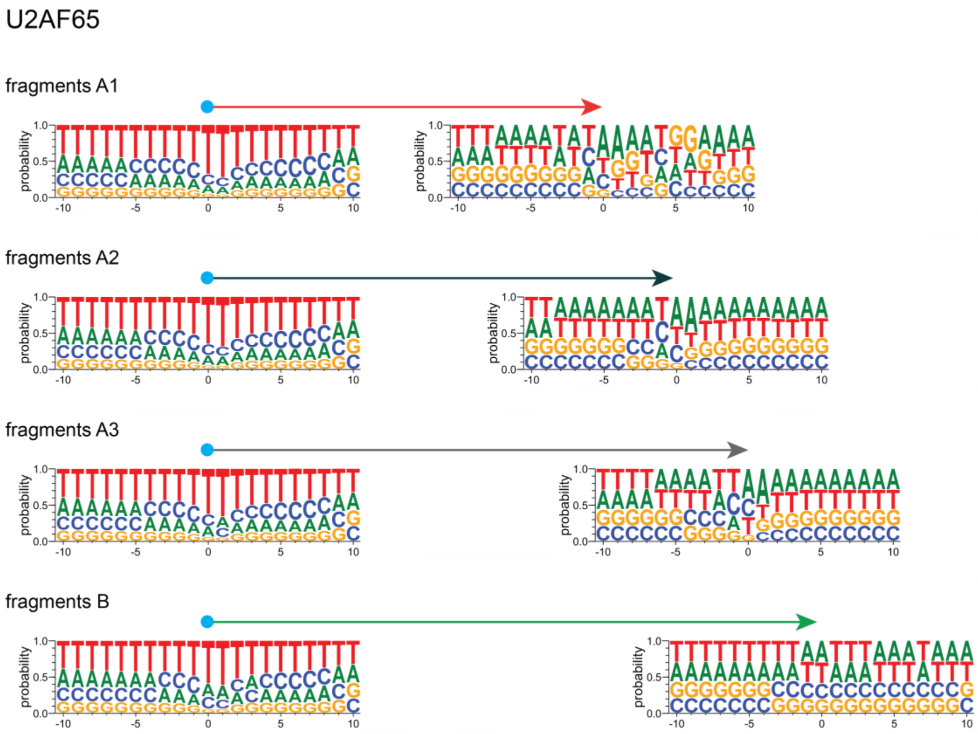
U2AF65
fragments A1
fragments A2
fragments A3
fragments B
probability

e

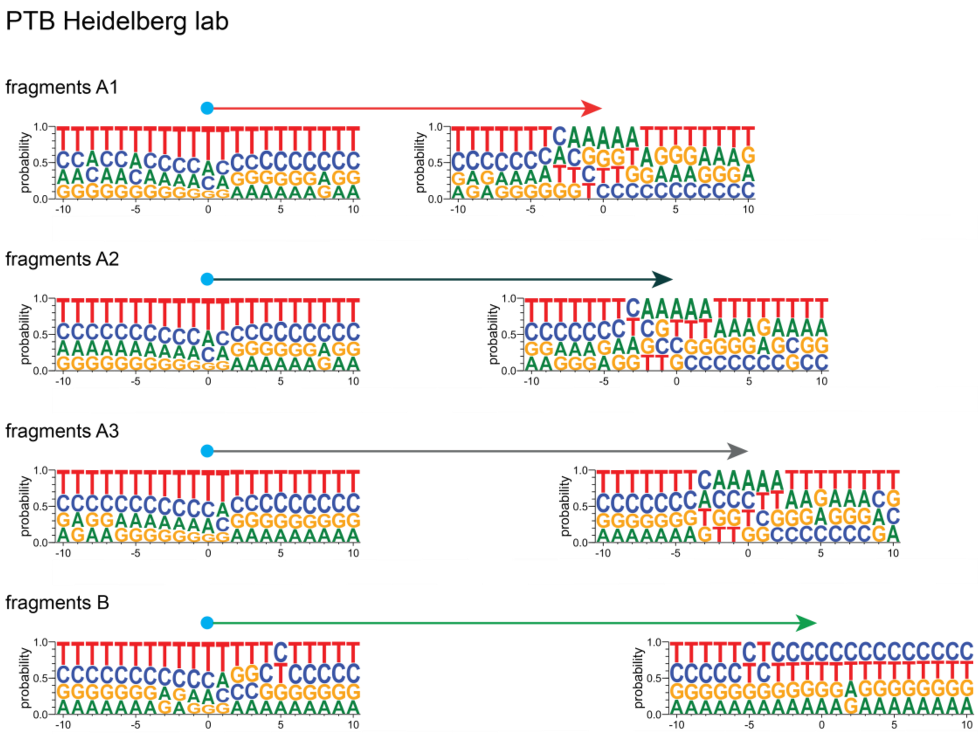
PTB Heidelberg lab
fragments A1
fragments A2
fragments A3
fragments B
probability

f

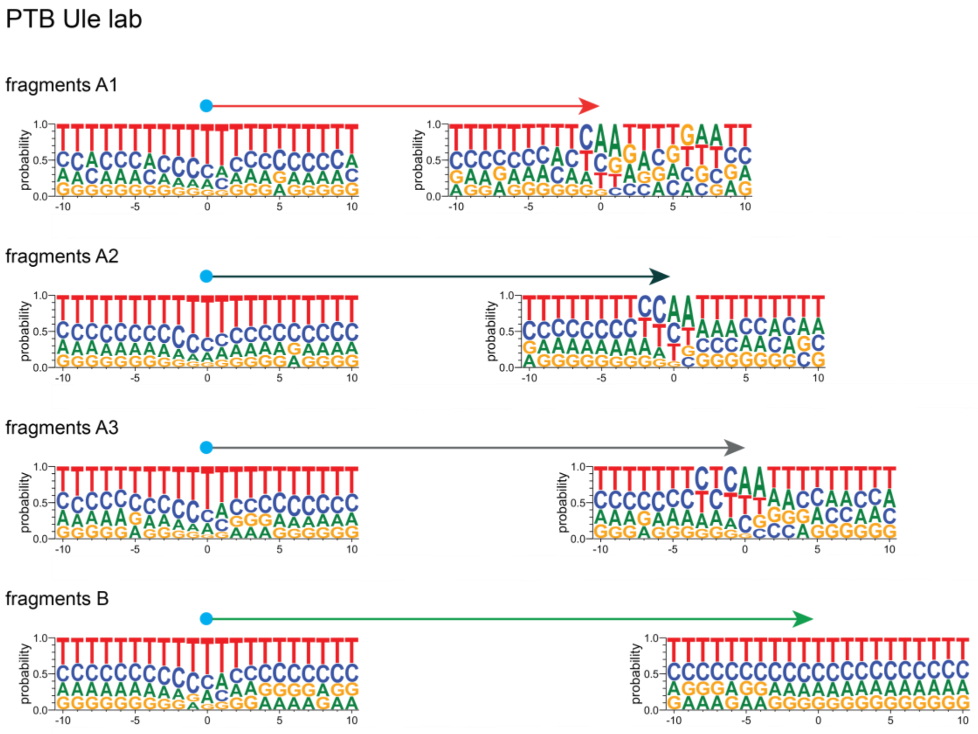
PTB Ule lab
fragments A1
fragments A2
fragments A3
fragments B
probability

g

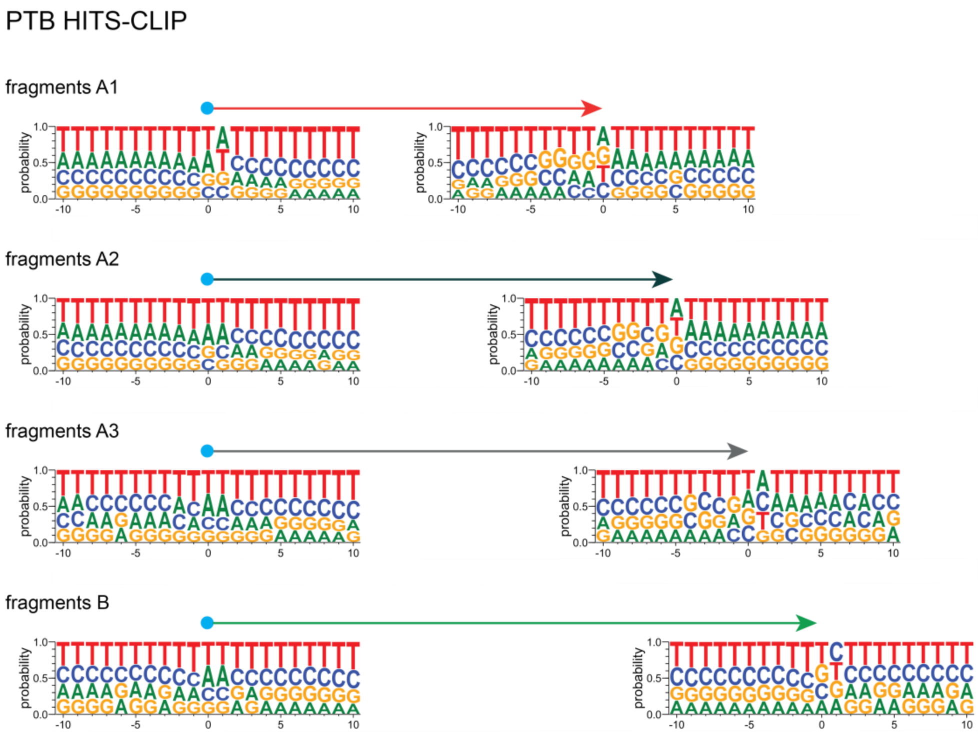

h

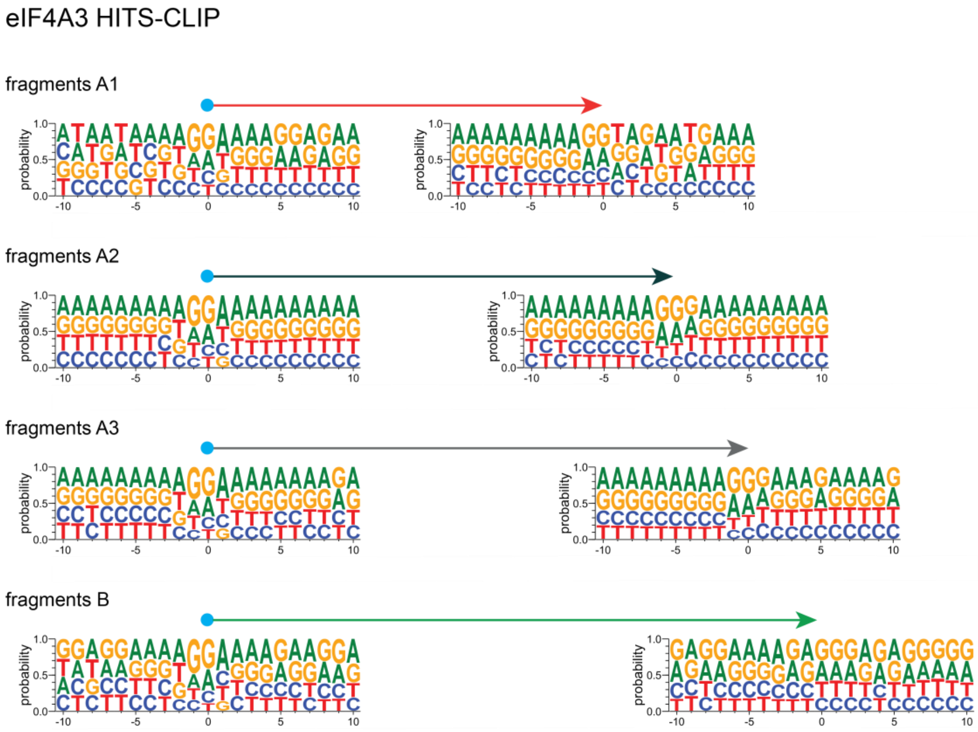

Supplementary Figure 5 Nucleotide compositions around start and end sites of CLIP fragments. Fragments were divided into separate categories as described in **Figure 2a** and **Supplementary Table**

2. The nucleotide composition was examined around start and end of fragments of different lengths at the predominant binding sites for libraries of (**a**) eIF4A3, (**b**) SRSF3, (**c**) hnRNP L, (**d**) U2AF65, (**e**) PTB Heidelberg lab, (**f**) PTB Ule lab, (**g**) PTB HITS-CLIP, and (**h**) eIF4A3 HITS-CLIP.

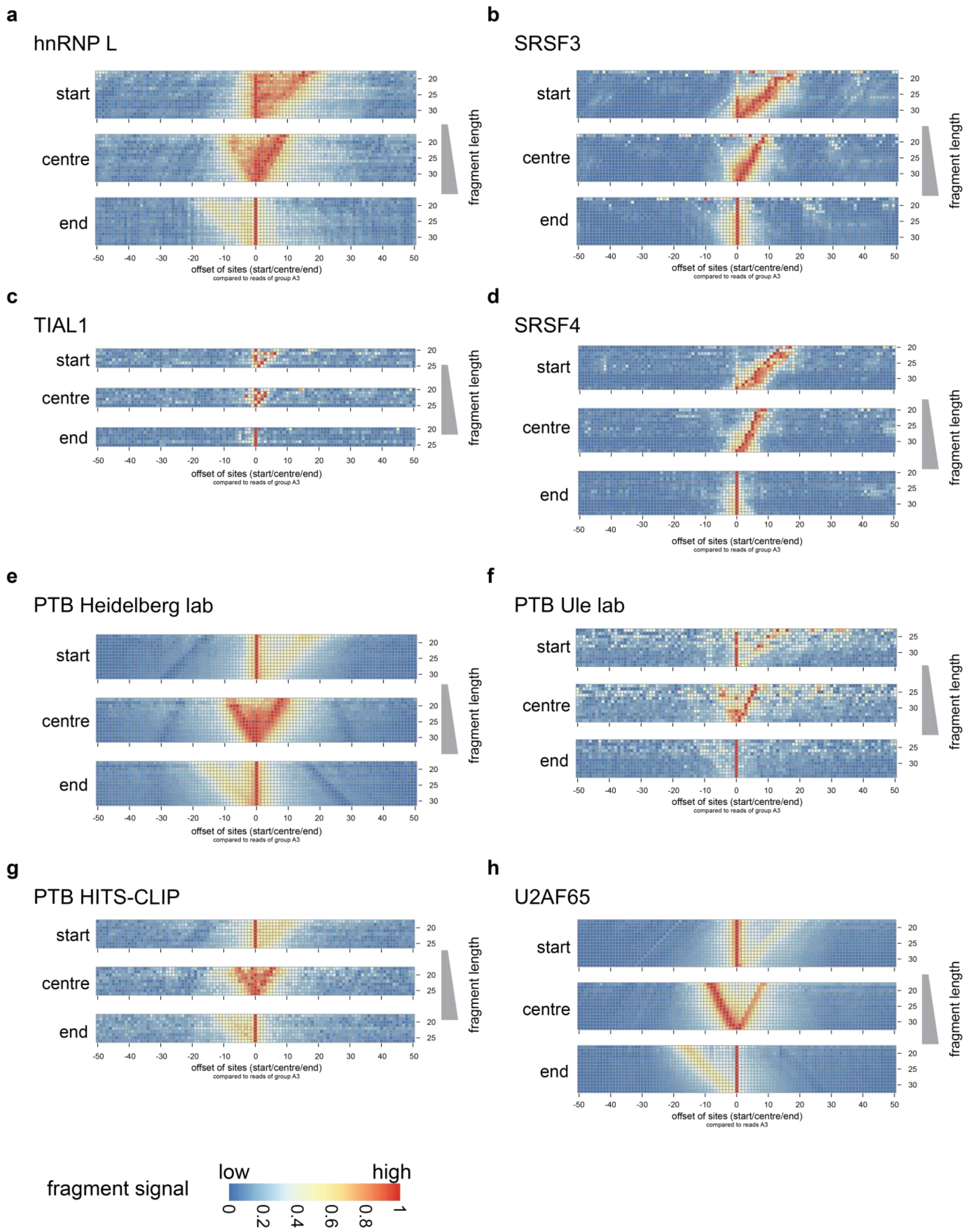

Supplementary Figure 6 High resolution read overlap heatmaps for the start, centre and end positions of fragments. The high-resolution read overlap heatmaps for the different RBPs are shown for (**a**) hnRNP L (mean overlap start site ratio 0.82), (**b**) SRSF3 (0.40), (**c**) TIAL1 (NA), (**d**) SRSF4 (0.32), (**e**) PTB Heidelberg lab (0.96), (**f**) PTB Ule lab (0.78), (**g**) PTB HITS-CLIP (1.04), and (**h**) U2AF65 (1.25). The high-resolution read overlap heatmaps were generated as described in **Figure 4**, with the

difference that completely sequenced fragments of group A3 are used for defining the reference 0-positions of either the start site (top panel), the centre (middle panel) and the end (bottom panel) of the fragments. The distribution of the fragment lengths are given in **Supplementary Figure 1** and the fragment length for A3 in **Supplementary Table 2**.

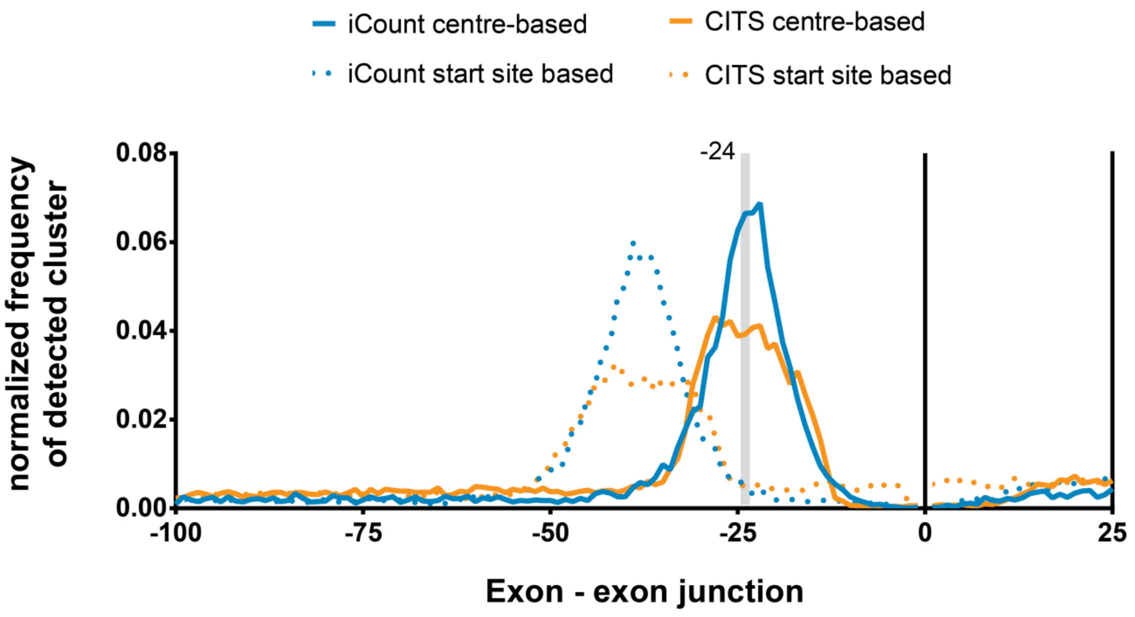

Supplementary Figure 7 Distribution of cross-linking clusters for the eIF4A3 iCLIP library relative to exon-exon junctions. The distribution of cross-linking clusters based on the start sites of eIF4A3 iCLIP fragments are shifted to positions upstream of the known region of RNA binding. The distribution of cross-linking clusters was determined by the iCount[1,2] and the cross-linking induced truncation sites (CITS)[3] methods resulting in almost concordant results with a slight increase of the signal obtained with the iCount algorithm. The use of the centre of completely sequenced iCLIP fragments as an input for these two methods reflected the known eIF4A3 binding site at a position approximately 24 nts upstream of the exon-exon junction.

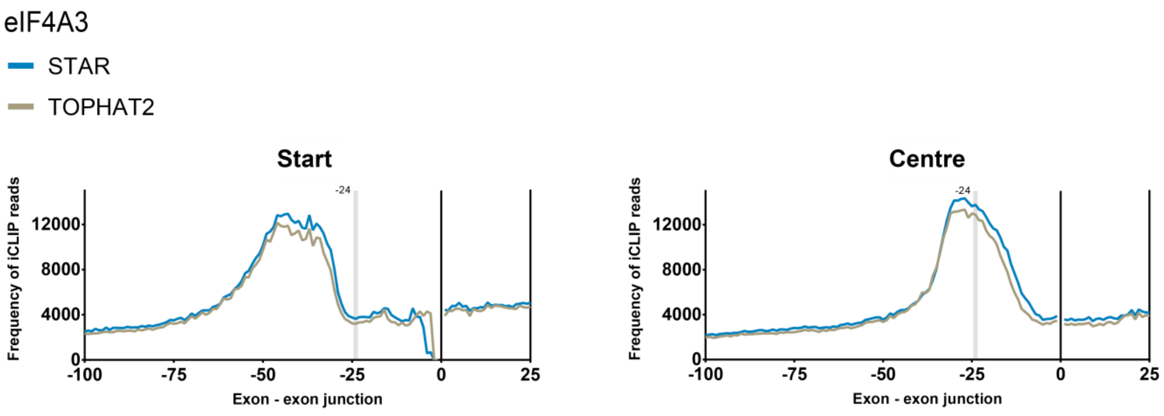

Supplementary Figure 8 Read distribution maps for eIF4A3 iCLIP library using different mapping programs. The graphs show the distributions of the entire population of fragments relative to exon-exon junctions. The left and right columns show the mapping of, respectively, the start and the centre positions of the iCLIP fragments. In both methods, the use of the centre reflected the known eIF4A3 binding site at a position approximately 24 nts upstream of the exon-exon junction.

Supplementary Table 1: Mapping statistics for HITS-CLIP and iCLIP libraries

	Lab of CLIP experiment	Total reads	Reads after 3´Solexa primer removal	Uniquely mapped reads	Reads after duplicate removal and quality filtering	Reads with deletions [%] (after quality filtering)
**eIF4A3**	Hentze/Kulozik, Heidelberg, Germany	29,781,052	28,329,506	8,165,916	1,969,800	1.7
**eIF4A3 HITS-CLIP**	Le Hir, Paris, France	33,670,095	32,539,607	14,141,213	13,906,546*	10.2
**SRSF3 (Mouse)**	Neugebauer, Dresden, Germany	8,224,275	7,412,024	5,080,281	974,855	0.3
**SRSF4 (Mouse)**	Neugebauer, Dresden, Germany	808,753	645,652	430,766	156,509	0.2
**PTB Heidelberg lab**	Hentze/Kulozik, Heidelberg, Germany	23,501,063	22,691,882	10,162,247	5,176,511	2.3
**PTB Ule lab**	Ule, London, United Kingdom	1,317,861	1,286,317	579,377	550,896	8.4
**PTB HITS-CLIP**	Zhang, Hubei, China	3,713,373	2,553,365	969,918	956,282*	2.7
**U2AF65**	Ule, London, United Kingdom	15,503,088	15,195,548	10,233,940	8,958,729	5.4
**hnRNP L**	Bindereif, Giessen, Germany	13,315,681	12,753,821	5,189,146	755,762	0.9
**TIAL1**	Ule, London, United Kingdom	2,537,210	2,209,931	1,368,141	69,553	0.2

*in HITS-CLIP libraries there is no random barcode to evaluate the duplicates

Supplementary Table 2: Fragment length sub-groups of the different HITS-CLIP and iCLIP libraries

	A1 [nts]	A2 [nts]	A3 [nts]	B [nts]
**eIF4A3**	17-24	25-32	33-39	≥ 42
**eIF4A3 HITS-CLIP**	17-35	36-54	55-77	≥ 80
**SRSF3 (Mouse)**	17-24	25-32	33-38	≥ 41
**SRSF4 (Mouse)**	17-24	25-33	34-42	≥ 45
**PTB Heidelberg lab**	17-23	24-31	32-39	≥ 42
**PTB Ule lab**	17-29	30-34	35-38	≥ 41
**PTB HITS-CLIP**	17-22	23-26	27-33	≥ 36
**U2AF65**	17-24	25-32	33-38	≥ 41
**hnRNP L**	17-24	25-32	33-38	≥ 41
**TIAL1**	17-21	22-25	26-28	≥ 31

Supplementary Table 3: Overlap start site ratio for coinciding start sites in HITS-CLIP and iCLIP libraries

	Reference group B			Reference group A3			Suggested analysis for binding site assignment
	Overlap start site ratio			Overlap start site ratio			
	median (flank 5 nts)	mean (flank 5 nts)	SD	median (flank 5 nts)	mean (flank 5 nts)	SD	
**eIF4A3**	0.91	0.88	0.08	0.97	0.97	0.05	centre-based
**eIF4A3 HITS-CLIP***	0.86	0.83	0.05	0.84	0.81	0.11	centre-based
**SRSF3 (Mouse)**	0.37	0.43	0.17	0.37	0.40	0.13	centre-based
**SRSF4 (Mouse)**	0.49	0.50	0.12	0.29	0.32	0.09	centre-based
**PTB Heidelberg lab**	0.96	0.92	0.09	0.98	0.96	0.06	centre-based
**PTB Ule lab**	0.89	0.89	0.18	0.78	0.78	0.15	centre-based
**PTB HITS-CLIP***	0.96	0.95	0.05	1.03	1.04	0.06	centre-based
**U2AF65**	1.33	1.31	0.16	1.28	1.25	0.09	start site based
**hnRNP L**	0.76	0.77	0.05	0.83	0.82	0.04	centre-based
**TIAL1****	1.08	1.04	0.15	NA	NA	NA	NA

*for HITS-CLIP libraries the use of the centre position is the default mode of analysis. In case of eIF4A3 HITS-CLIP the number of reads of group B is too small for reliable quantification of the read overlap heatmaps (see **Supplementary Fig. 1**). We therefore used fragments of length 33-39 nts as a reference in **Figure 6** and fragments of length 42-80 nts as reference in **Supplementary Figure 4**.
for TIAL1 the number of reads of group A is too small for reliable quantification of the read overlap heatmaps (see **Supplementary Fig. 1)

Supplementary Table 4: Influence on the overlap start site ratio by changing bin size and read number parameters

	Reads per bin	bin size [nts]	total number of bins	selected bins with at least 20/50 reads per bin	Reads in selected bins	Overlap start site ratio		
						Median (flank 5 nts)	Mean (flank 5 nts)	SD
eIF4A3	20	100	509,477	12,874	669,338	0.92	0.89	0.08
		300	358,376	16,704	920,732	0.90	0.87	0.09
		1000	245,877	18,674	1,191,740	0.90	0.86	0.10
	50	100	509,477	3,027	386,982	0.92	0.90	0.05
		300	358,376	4,151	552,791	0.91	0.88	0.08
		1000	245,877	5,647	802,028	0.90	0.87	0.09
U2AF65	20	100	2,070,558	77,089	3,444,163	1.43	1.39	0.15
		300	1,344,973	96,330	4,823,044	1.43	1.39	0.17
		1000	788,720	104,036	6,368,672	1.45	1.41	0.17
	50	100	2,070,558	16,483	1,665,998	1.30	1.27	0.14
		300	1,344,973	26,092	2,712,541	1.33	1.31	0.16
		1000	788,720	37,460	4,324,637	1.37	1.36	0.17

Supplementary Table 5: Comparison of start and centre fragment clusters for the iCLIP libraries in which the analysis mode should be centre-based

	fragments of group A			fragments of group B		
	Used start cluster within 25 nts of centre cluster (all start cluster)	Used centre cluster within 25 nt of start cluster (all centre cluster)	Median offset (start to centre cluster midpoints) [nts]	Used start cluster within 25 nts of centre cluster (all start cluster)	Used centre cluster within 25 nt of start cluster (all centre cluster)	Median offset (start to centre cluster midpoints) [nts]
**eIF4A3**	21,504 (24,713)	21,583 (25,282)	15	12,569 (14,803)	12,590 (14,957)	21
**SRSF3 (Mouse)**	5,212 (5,853)	5,216 (5,916)	14	5,015 (5,547)	5,006 (5,653)	21
**SRSF4 (Mouse)**	962 (1,108)	960 (1,113)	14	1,035 (1,191)	1,036 (1,211)	23
**PTB Heidelberg lab**	62,897 (69,911)	63,048 (70,799)	14	32,673 (36,077)	32,679 (36,206)	21
**PTB Ule lab**	3,151 (3,508)	3,166 (3,574)	15	11,469 (12,299)	11,475 (12,364)	21
**hnRNP L**	4,801 (5,400)	4,810 (5,460)	12	4,442 (5,098)	4,441 (5,230)	20

Supplementary References

1. Konig, J. *et al*. iCLIP reveals the function of hnRNP particles in splicing at individual nucleotide resolution. *Nat. Struct. Mol. Biol.* **17**, 909-15 (2010).
2. Sugimoto, Y. *et al*. Analysis of CLIP and iCLIP methods for nucleotide-resolution studies of protein-RNA interactions. *Genome Biol.* **13**, R67 (2012).
3. Weyn-Vanhentenryck, S.M. *et al*. HITS-CLIP and integrative modeling define the Rbfox splicing-regulatory network linked to brain development and autism. *Cell Rep.* **6**, 1139-52 (2014).
